# Association of social isolation and loneliness with risk of incident hospital-treated infections: an analysis of data from the UK Biobank and Finnish Health and Social Support studies

**DOI:** 10.1016/S2468-2667(22)00253-5

**Published:** 2023-01-17

**Authors:** Marko Elovainio, Kaisla Komulainen, Pyry N Sipilä, Laura Pulkki-Råback, Laura Cachón Alonso, Jaana Pentti, Solja T Nyberg, Sakari Suominen, Jussi Vahtera, Jari Lipsanen, G David Batty, Christian Hakulinen, Mika Kivimäki

**Affiliations:** a Department of Psychology and Logopedics, Faculty of Medicine, University of Helsinki, Helsinki, Finland; b Clinicum, Faculty of Medicine, University of Helsinki, Helsinki, Finland; c Finnish Institute for Health and Welfare, Helsinki, Finland; d Department of Public Health, University of Turku, Turku, Finland; e Centre of Population Health Research, Turku University Hospital, Turku, Finland; f School of Health Sciences, University of Skövde, Skövde, Sweden; g Department of Epidemiology and Public Health, University College London, London, UK; h Department of Mental Health of Older People, Faculty of Brain Sciences, University College London, London, UK

## Abstract

**Background:**

Although loneliness and social isolation have been linked to an increased risk of non-communicable diseases such as cardiovascular disease and dementia, their association with the risk of severe infection is uncertain. We aimed to examine the associations between loneliness and social isolation and the risk of hospital-treated infections using data from two independent cohort studies.

**Methods:**

We assessed the association between loneliness and social isolation and incident hospital-treated infections using data for participants from the UK Biobank study aged 38–73 years at baseline and participants from the nationwide population-based Finnish Health and Social Support (HeSSup) study aged 20–54 years at baseline. For inclusion in the study, participants had to be linked to national health registries, have no history of hospital-treated infections at or before baseline, and have complete data on loneliness or social isolation. Participants with missing data on hospital-treated infections, loneliness, and social isolation were excluded from both cohorts. The outcome was defined as a hospital admission with a primary diagnosis of infection, ascertained via linkage to electronic health records.

**Findings:**

After exclusion of 8·6 million participants for not responding or not providing appropriate consent, the UK Biobank cohort consisted of 456 905 participants (249 586 women and 207 319 men). 26 860 (6·2%) of 436 001 participants with available data were reported as being lonely and 40 428 (9·0%) of 448 114 participants with available data were socially isolated. During a median 8·9 years (IQR 8·0–9·6) of follow-up, 51 361 participants were admitted to hospital due to an infectious disease. After adjustment for age, sex, demographic and lifestyle factors, and morbidities, loneliness was associated with an increased risk of a hospital-treated infection (hazard ratio [HR] 1·12 [95% CI 1·07–1·16]), whereas social isolation was not (HR 1·01 [95% CI 0·97–1·04]). Of 64 797 individuals in the HeSSup cohort, 18 468 (11 367 women and 7101 men) were eligible for inclusion. 4466 (24·4%) of 18 296 were lonely and 1776 (9·7%) of 18 376 socially isolated. During a median follow-up of 10·0 years (IQR 10·0–10·1), 814 (4·4%) participants were admitted to hospital for an infectious disease. The HRs for the HeSSup study replicated those in the UK Biobank (multivariable-adjusted HR for loneliness 1·32 [95% CI 1·06–1·64]; 1·08 [0·87–1·35] for social isolation).

**Interpretation:**

Loneliness might increase susceptibility to severe infections, although the magnitude of this effect appears modest and residual confounding cannot be excluded. Interventional studies are required before policy recommendations can advance.

**Funding:**

Academy of Finland, the UK Medical Research Council, and Wellcome Trust UK.

## Introduction

Loneliness refers to the discrepancy between the desired and actual quality and quantity of social relationships that a person experiences, whereas social isolation is denoted by an absence of contacts within a social network or community.^1^, ^2^ Loneliness and social isolation have been associated with an increased risk of various non-communicable diseases such as depression,^3^ cardiovascular diseases,^4^ and dementia.^5^ However, little is known about their association with infectious diseases.

Some plausible mechanisms connect loneliness and social isolation with an increased risk of infections. Generally, lonely and socially isolated people experience more psychological stress^6^, ^7^ and have a less healthy lifestyle^8^ than their socially active counterparts and might therefore be more susceptible to infections when they encounter a pathogen. Studies have suggested that psychological stress increases the likelihood of developing an infection after standard viral exposure, delays recovery from infections, and renders an individual more susceptible to the reactivation of latent viruses within the body (eg, herpes viruses).^9^, ^10^ Viral-challenge experiments comparing groups with and without stressors such as loneliness have suggested that long-term stress reduces the sensitivity of immune cells to glucocorticoids (which normally downregulate the inflammatory response), weakens resistance to upper respiratory infections, and increases illness symptoms.^9^, ^10^, ^11^, ^12^ Observational cohort studies have linked loneliness and social isolation to prolonged systemic inflammation and an increased risk of hospital admission due to upper respiratory tract infections, although the evidence is not entirely consistent.^11^, ^12^

**Research in context**

**Evidence before this study**

Both loneliness (discrepancy between the desired and actual quality and quantity of social relationships, irrespective of their number) and social isolation (low number of social relations) have been associated with an increased risk of non-communicable conditions, such as cardiovascular disease and dementia; however, their association with risk of severe infection is unclear. To identify research on the association of loneliness and social isolation with infectious diseases, we searched PubMed for studies published in English from inception up to Feb 28, 2022, using the following search terms (in title): social support AND infect* (72 publications); social relations AND infect* (1); social networks AND infect* (35); social isolation AND infect* (6); loneliness AND infect* (6); loneliness AND infla* (16); and social isolation AND infla* (16). We found no large-scale studies on the risk of severe infectious diseases among lonely or socially isolated individuals. At least one study linked loneliness to increased risk of hospital admission due to respiratory tract infections, but this was not confirmed in another study. Lonely people experience more psychological stress and experimental and observational studies suggested that psychological stress might increase susceptibility to infections. On the other hand, the reduced human contacts of socially isolated individuals could decrease exposure to human-to-human transmitted infectious pathogens.

**Added value of this study**

We used individual-level data from two large, independent cohort studies (the UK Biobank and Finnish Health and Social Support studies) linked to hospital records. After considering confounding factors including socioeconomic status, extant illness, and ethnicity, loneliness was associated with a modest increase in risk of hospital-treated infections in both cohorts. The association was also replicated in population subgroups, and we found no evidence of reverse causality. By contrast, the association between social isolation and hospital-treated infections was not robust and attenuated to null after adjustment for confounders.

**Implications of all the available evidence**

Our findings support the hypothesis that loneliness might increase susceptibility to severe infectious diseases. The absence of a similar association for social isolation is plausible given the reduced number of human contacts and thus lower exposure to human-to-human transmission of pathogens among socially isolated individuals, and because social isolation is not necessarily combined with perceived stress. Interventional studies are required before policy recommendations can advance.

However, loneliness and social isolation can also affect exposure to infectious pathogens in protective ways. The reduced human contact of socially isolated individuals could potentially decrease their exposure to human-to-human transmitted pathogens. Moreover, reverse causation is also possible (ie, loneliness and social isolation could be consequences rather than causes of the disease). Because many infectious diseases spread through social contact, prevention strategies seek to minimise social relations (eg, physical–social isolation, physical distancing, and quarantine of infected patients until recovery from the disease). Patients with specific chronic infectious diseases, such as HIV and tuberculosis, might experience stigma, further increasing the likelihood of social exclusion.^13^ Infections can also adversely affect social relations via sickness behaviour, including loss of appetite, sleepiness, fatigue, increased pain, anhedonia, and social withdrawal.^14^

To better understand associations of loneliness and social isolation with the risk of infectious diseases, we examined whether lonely and socially isolated individuals were at increased risk of hospital admission due to more than 900 infectious diseases across two well characterised cohort studies, and investigated whether loneliness and social isolation were more likely to precede or follow infectious diseases.

## Methods

### Study design and population

We assessed the association of loneliness and social isolation with hospital-treated infectious diseases using data from the prospective UK Biobank cohort study,^15^ and replicated this analysis using data from an independent cohort, the nationwide population-based Finnish Health and Social Support (HeSSup) study.^16^ Baseline data for the UK Biobank were collected between 2006 and 2010 in 22 research assessment centres across the UK. We included participants aged 38–73 years, who were linked to national health registries, had no history of hospital-treated infections at or before baseline, and had complete data on loneliness or social isolation. The HeSSup study comprised a random sample of individuals in Finland aged 20–54 years.^16^ We included individuals from the HeSSup study with available data on loneliness or social isolation who were linked to national health registries. The HeSSup study included repeated assessments of loneliness and social isolation (in 1998 and 2003), which allowed evaluation of reverse causality. We excluded participants with missing data on hospital-treated infections, loneliness, and social isolation from both cohorts. The outcome of interest was defined as hospital admissions with a primary diagnosis of infection, ascertained via linkage to electronic health records.

All participants provided written informed consent for the baseline assessments and for registry linkage. The UK Biobank was approved by the National Health Service National Research Ethics Service (11/NW/0382), and the HeSSup study by the ethics committee of Turku University Central Hospital and the Finnish Population Register Centre (VRK 2605/410/14).

### Procedures

In the UK Biobank, loneliness was assessed by asking two questions: “Do you often feel lonely?” (no, 0; yes, 1) and “How often are you able to confide in someone close to you?” (0, almost daily–once every few months; 1, never or almost never). We defined a person as lonely only if they responded positively to both questions. In the sensitivity analysis, we used a single-item measure (“Do you often feel lonely?” Yes or no) to measure loneliness, as such single-item measures are highly correlated with the UCLA loneliness scale, the most commonly used multi-item measure of loneliness in population surveys.^17^ In the UK Biobank, social isolation was assessed by asking three questions:^15^ (1) “Including yourself, how many people live in your household? Include those who usually live in the house such as students living away from home during term time, and partners in the armed forces or in professions such as pilots” (1 point for living alone); (2) “How often do you visit friends or family or have them visit you?” (1 point for less than one friend or family visit per month); and (3) “Which of the following (leisure or social activities) do you engage in once a week or more often? You may select more than one” (1 point for not participating in any social activities at least weekly). The sum of the responses to these three questions resulted in a scale ranging from 0 to 3. We classified respondents with 2 or 3 points as socially isolated. The loneliness and social isolation measures were dichotomised with no weighting of responses.

In the HeSSup study, we constructed binary variables of loneliness and social isolation measures to make the measures and distributions as comparable as possible with those in the UK Biobank. Loneliness was assessed by asking “Do you currently feel lonely?” (Yes, very much so; yes, to some extent; no). We dichotomised responses to this question into yes versus no. The social isolation measure included four items of the longer Social Support Questionnaire, reflecting different ways of receiving support.^18^ The respondents could choose one or more of six alternatives (husband, wife, or partner; some other relative; close friend; close co-worker; close neighbour; or someone else close). The responses to the items were combined so that each source of support contributed one point to the final social support score (range 0–20).^18^ We used dichotomised scores in our analyses (0–6, socially isolated; 7–20, not isolated). Additional references and a description of validity issues are provided in the appendix (p 2).

Data regarding sex were acquired from central registry at recruitment, but in some cases were updated by the participant (categories were male and female). We selected baseline covariates based on factors used in previous studies in the field (appendix p 2), including demographic characteristics such as age, sex, and ethnicity, which can act as confounders, and other factors that can act as confounders, mediators, or both (appendix p 3). In the UK Biobank, baseline covariates (self-reported unless otherwise specified, as in the HeSSup study) were sex, age, ethnicity (White or non-White), education (low [no secondary education], intermediate [secondary education], or high [university degree]), the Townsend deprivation index (a continuous measure of neighbourhood deprivation), chronic diseases (self-reported long-standing illness without any specific diagnosis), current smoking (yes or no), physical activity (moderate and vigorous physical activity five or more times a week *vs* other), frequency of alcohol intake (three or four times a week or more *vs* once or twice a week or less), BMI (kg/m^2^), depressed mood in the past 2 weeks (Patient Health Questionnaire classified as low [not at all, several days] and high [more than half of my days, nearly every day]), and C-reactive protein (mg/L). Using linked electronic health records, we assessed physical conditions that, according to the US Centers for Disease Prevention and Control (2017), increase the risk of infectious diseases (a list of conditions with International Classification of Diseases 10th Revision [ICD-10] codes is provided in the appendix p 2).

In the HeSSup cohort, all covariates were self-reported and included age, sex (categories male and female), education (low [no occupational education], intermediate [vocational education], or high [university education]), current smoking (yes or no), alcohol consumption (none or moderate [1 to 21 units], or heavy [>21 units per week]), BMI (kg/m^2^), and depressed mood (moderate depression based on Beck Depression Inventory [score >18]). Physical activity was assessed as Metabolic Equivalent of Tasks^19^ (METs) and was dichotomised on the basis of median split (high, 3·6 or more; low, <3·6 METs; this produced a broadly similar distribution to that in the UK Biobank study and a threshold close to that recommended previously).^20^

The UK Biobank participants were linked to the Hospital Episode Statistics Admitted Patient Care (England), the Scottish Morbidity Records General/Acute Inpatient and Day Case Admissions (Scotland), and the Patient Episode Database (Wales) until Feb 7, 2018. HeSSup participants were linked to the Finnish National Registry for Hospitalisations until Dec 31, 2012. In both studies, we retrieved primary diagnoses of infectious diseases from inpatient hospital discharge information using ICD-10 codes.^21^ We classified hospital-treated infectious diseases according to 925 ICD-10 codes (appendix pp 5–9). For comparison, we examined the associations of loneliness and social isolation with other broad disease categories including cancers; diseases of the endocrine, circulatory, respiratory, digestive, musculoskeletal, genitourinary, and nervous systems; diseases of the blood, eye, ear, and skin; and mental and behavioural disorders.

### Statistical analysis

On the basis of a log-rank test and assuming a 5% confidence level and a statistical power of 80%, the minimum sample size for the detection of a small relative risk of 1·1 was 15 055 for loneliness and 14 925 for social isolation. After we assessed the proportional hazards assumption (appendix pp 9–10), we used Cox proportional hazards models to estimate hazard ratios (HRs) and 95% CIs separately for the associations of loneliness and social isolation with the first hospital-treated infection episode. Participants with a hospital-treated infection at or before baseline were excluded before the analysis. Follow-up was from study entry until the first hospital episode due to infection, death, or end of follow-up, whichever came first (no data were available on migration). These analyses applied to both the UK Biobank and HeSSup studies (HeSSup data did not include information on ethnicity, area deprivation, or C-reactive protein).

We examined the associations of loneliness and social isolation with hospital-treated infectious diseases separately in the following steps. First, the associations were tested in the UK BioBank cohort by adjusting HRs and 95% CIs for age, sex, and ethnicity. To examine whether the associations were observable in subgroups, we conducted analyses stratified by sex, age, education, C-reactive protein, long-term disease status, and depressed mood at baseline. These variables were selected because they are potential effect modifiers and were used in stratified analyses in previous UK Biobank studies on loneliness.^8^, ^22^ The interaction effect was tested by adding interaction terms into each model.

Second, we performed stepwise multivariable analyses in the UK BioBank cohort to test the extent to which the associations were independent of baseline covariates, and whether the multivariable-adjusted results were replicable in the first 3 years of follow-up and from year 3 onwards. All models included loneliness or social isolation as the exposure and covariates were added as follows. Model 1 included age and sex. In addition to age and sex, other models included ethnicity (Model 2); education and the Townsend deprivation index (Model 3); smoking, alcohol consumption, physical activity, and BMI (Model 4); long-term illness (Model 5); C-reactive protein (Model 6); depressed mood (Model 7); and all the aforementioned covariates (Model 8). Given that the covariates can act as both as confounders and mediators, we interpreted the results cautiously and considered the association between loneliness or social isolation and infectious diseases independent of other factors only if the association remained significant after adjustment for the covariates. We calculated the percentage of excess risk attributable to covariates (PERM) for the associations of social isolation and loneliness with infections using the following formula:

PERM% = ([HR(age and sex) – HR(age, sex, and covariates adjusted)]/[HR(age and sex adjusted) – 1]) × 100.

Third, in sensitivity analyses of the UK BioBank cohort, we tested whether the associations were robust to the exclusion of participants with physical conditions that increase the risk of infectious diseases. To examine reverse causation in the HeSSup study, we tested whether infectious diseases at baseline were associated with loneliness or social isolation at follow-up among those who did not report these exposures at baseline. The exposure was a hospital-treated infectious disease and the outcome loneliness or social isolation. We included those with and without an infectious disease at baseline (the exposure) but excluded those who reported being lonely or isolated. Incident cases were those who had become lonely or socially isolated at follow-up. To investigate disease specificity, we examined associations between loneliness or social isolation and other disease categories. In each step, participants with missing data on covariates were excluded from the analysis.

A two-sided p value of less than 0·05 was considered to indicate statistical significance. Because this was a hypothesis-testing study with multiple sensitivity analyses rather than an exploratory study with multiple independent tests, we did not correct for multiple testing.

In the HeSSup study, the analyses were done in two steps, first adjusted for age and sex and second adjusted for age, sex, education, alcohol consumption, smoking status, physical activity, and depressive symptoms.

We did all data analyses in R (version 4.1.1) between December, 2021, and January, 2022. The code for the analyses is available in the appendix (pp 11–44).

### Role of the funding source

The funders of the study had no role in study design, data collection, data analysis, data interpretation, or the writing of the report.

## Results

**Figure 1:**
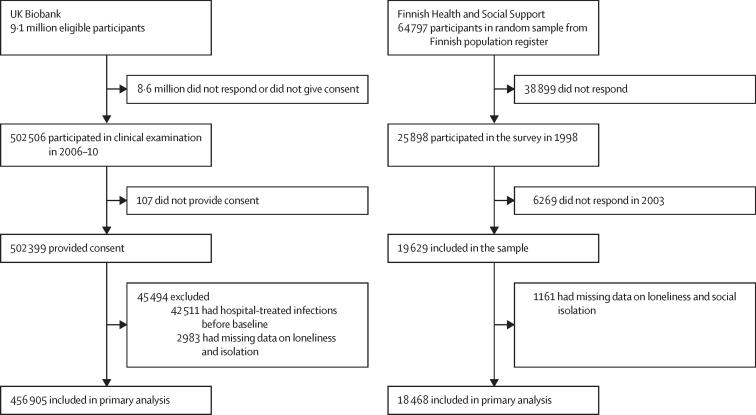
Study profiles

The UK Biobank cohort comprised 9·1 million eligible individuals, of whom 8·6 million did not respond or did not provide consent. Therefore, at baseline, the UK biobank cohort comprised 502 506 participants aged 38–73 years (5·5% of the total UK Biobank cohort), of whom 456 905 (90·9%) were linked to national health registries, had no history of hospital-treated infections at or before baseline, and had complete data on loneliness or social isolation (figure 1). The UK Biobank sample included 249 586 women (54·6%) and 207 319 men (45·4%), with a mean baseline age of 56·5 years (SD 8·1; table).

**Table:** Characteristics of the participants from the UK Biobank study (baseline 2006–10) and the HeSSup study, Finland (baseline 1998)

	**UK Biobank (n=456 905)**	**HeSSup (n=18 468)**
**Sex**		
Female	249 586 (54·6%)	11 367 (61·5%)
Male	207 319 (45·4%)	7101 (38·5%)
**Age, years**		
Mean (SD)	56·5 (8·1)	42·5 (11·4)
Median (IQR)	58·0 (50·0–63·0 )	45·0 (35·0–55·0 )
**Age groups, years**		
0–49	107 242 (23·5%)	4588 (24·8%)
50–59	152 895 (33·5%)	8787 (47·6%)
60 or older	196 768 (43·1%)	5093 (27·6%)
**Ethnicity**		
White	431 725/455 363 (94·8%)	..
Other	23 638/455 363 (5·2%)	..
Missing	1542 (0·3%)	..
**Education**		
Low	74 631/448 320 (16·6%)	4175/18 370 (22·7%)
Intermediate	224 141/448 320 (50·0%)	9185/18 370 (50·0%)
High	149 548/448 320 (33·4%)	5010/18 370 (27·3%)
Missing	8585 (1·9%)	98 (0·5%)
**Townsend index tertile**		
Bottom	154 786/456 341 (33·9%)	..
Intermediate	153 185/456 341 (33·6%)	..
Top	148 370/456 341 (32·5%)	..
Missing	564 (0·1%)	..
**Alcohol consumption**		
Low	255 247/456 556 (55·9%)	6030/18 415 (32·7%)
High	201 309/456 556 (44·1%)	12 385/18 415 (67·3%)
Missing	349 (0·1%)	53 (0·3%)
**Current smoker**		
No	408 708/455 295 (89·8%)	5515/8898 (62·0%)
Yes	46 587/455 295 (10·2%)	3383/8898 (38·0%)
Missing	1610 (0·4%)	9570 (51·8%)
**Physical activity**		
Low	250 795/420 802 (59·6%)	9786/18 345 (53·3%)
High	170 007/420 802 (40·4%)	8559/18 345 (46·7%)
Missing	36 103 (7·9%)	123 (0·7%)
**BMI category**		
Normal weight	149 915/446 101 (33·6%)	9695/18 346 (52·8%)
Overweight	190 555/446 101 (42·7%)	6195/18 346 (33·8%)
Obese	105 631/446 101 (23·7%)	2456/18 346 (13·4%)
Missing	10 805 (2·4%)	122 (0·7%)
**BMI**		
Mean (SD)	27·3 (4·7)	25·4 (4·5)
Missing	10 805 (2·4%)	122 (0·7%)
Depressed mood*		
Low	415 836/436 658 (95·2%)	17 135/18 097 (94·7%)
High	20 822/436 658 (4·8%)	962/18 097 (5·3%)
Missing	20 247 (4·4%)	371 (2·0%)
**Long-term illness**		
No	224 804/443 289 (50·7%)	6177/18 362 (33·6%)
Yes	218 485/443 289 (49·3%)	12 185/18 362 (66·4%)
Missing	13 616 (3·0%)	106 (0·6%)
**High C-reactive protein (>3 mg/L)**		
No	333 223/426 655 (78·1%)	..
Yes	93 432/426 655 (21·9%)	..
Missing	30 250 (6·6%)	..
**Socially isolated**		
No	407 686/448 114 (91·0%)	16 600/18 376 (90·3%)
Yes	40 428/448 114 (9·0%)	1776/18 376 (9·7%)
Missing	8791 (1·9%)	92 (0·5%)
**Feeling lonely**		
No	409 141/436 001 (93·8%)	13 830/18 296 (75·6%)
Yes	26 860/436 001 (6·2%)	4466/18 296 (24·4%)
Missing	20 904 (4·6%)	172 (0·9%)
**Follow-up time from baseline, years**		
Mean (SD)	8·5 (1·9)	9·8 (1·3)
Median (IQR)	8·9 (8·0–9·6 )	10·0 (10·0–10·1 )

Data are n (%) or n/N (%), unless otherwise specified. HeSSup=Finnish Health and Social Support study.

* Based on the frequency of depressed mood in the previous 2 weeks from the Patient Health Questionnaire in the UK Biobank study and on the Beck Depression inventory with a cutoff of 18 or more in the HeSSup study.

**Figure 2:**
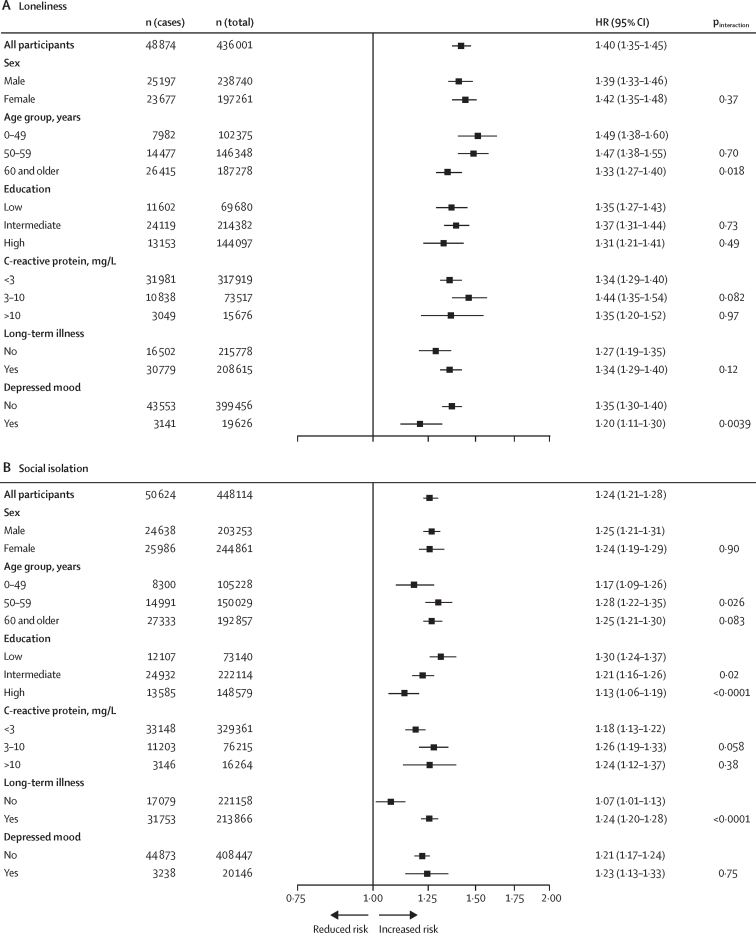
Associations of loneliness (A) and social isolation (B) with incident hospital-treated infections in different subgroups in the UK Biobank

26 860 (6·2%) of 436 001 participants with available data were classified as being lonely and 40 428 (9·0%) of 448 114 were classified as socially isolated (table). During a median 8·9 years (IQR 8·0–9·6) of follow-up (3 874 229 person-years at risk), we recorded 51 361 (11·2%) hospital-treated infections in 456 905 participants after the study baseline, and 24 296 (5·3%) all-cause deaths. The age, sex, and ethnicity-adjusted HR for the risk of hospital-treated infection was 1·40 (95% CI 1·35–1·45) for participants who were lonely versus those who were not (figure 2). This association was observed in analyses stratified by sex, age, education, C-reactive protein, chronic disease status, and depressed mood at baseline (figure 2).

**Figure 3:**
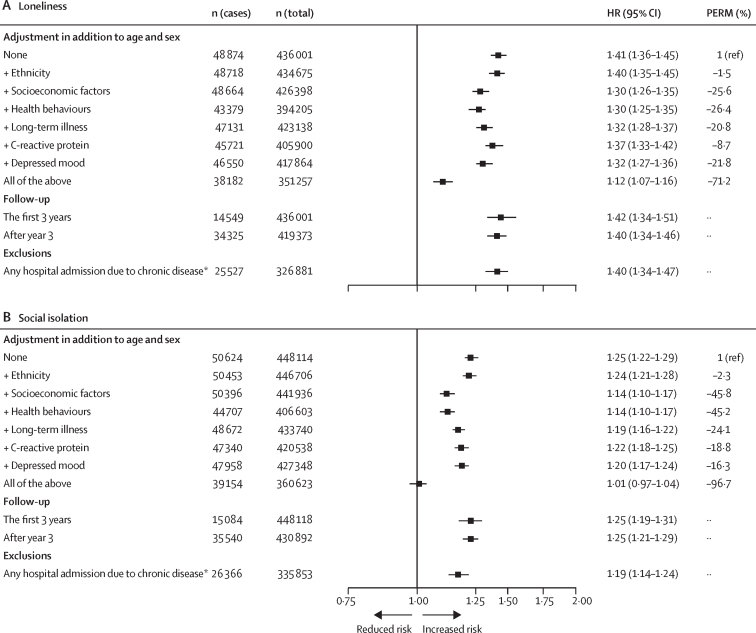
Associations of loneliness (A) and social isolation (B) with hospital-treated infections in the UK Biobank after multivariable adjustments, in different follow-up groups, and excluding those with hospital admissions due to diseases that could affect the risk of infections PERM=percentage of excess risk attributable to covariates. *Participants with cancer, chronic kidney disease, chronic lung diseases, dementia or other neurological diseases, diabetes, heart conditions, HIV infection, primary immune deficiency, liver disease, sickle cell disease, stroke or cerebrovascular disease, and substance use disorder at baseline or follow-up were excluded.

The association between loneliness and hospital-treated infections was robust to adjustment for age, sex, ethnicity, socioeconomic factors, health behaviours, long-term illness, C-reactive protein, and depressed mood (figure 3). After adjusting for all these factors, the HR for the risk of hospital-treated infections among participants who were classified as lonely versus those who were not was 1·12 (95% CI 1·07–1·16) (figure 3). Health behaviours (PERM, 26·4%), socioeconomic factors (25·6%), and depressed mood (21·8%) were the strongest contributors to the association. The association between loneliness and infections remained in all sensitivity analyses using a single-item measure of loneliness (appendix pp 46–51), was not dependent on follow-up time, and did not attenuate when those with a history of infection-related diseases were excluded. In the outcome-wide analysis, loneliness was also associated with hospital-treated mental and behavioural disorders and diseases of the respiratory, endocrine, nervous, and musculoskeletal systems (appendix p 51). There were no associations with other diseases or the associations were weak (relative risk <1·10 for diseases of the digestive and circulatory systems and diseases of the skin and eye and ear). When repeating the analyses using the single-item loneliness measure, the multivariable-adjusted risk of infectious disease was 1·11 times (95% CI 1·08–1·14) higher in participants with loneliness than in those not reporting loneliness.

The age, sex, and ethnicity-adjusted HR for the risk of hospital-treated infection among individuals who were socially isolated versus those who were not was 1·24 (95% CI 1·21–1·28; figure 2). The HRs in the subgroups ranged from 1·07 (95% CI 1·01–1·13) for participants without chronic conditions to 1·30 (1·24–1·37) for those with a low education level (figure 2). In the multivariable analyses including all covariates, the association between social isolation and hospital-treated infections attenuated to null (HR 1·01, 95% CI 0·97–1·04; figure 3).

The HeSSup study comprised 64 797 individuals in Finland aged 20–54 years.^16^ Of these, 25 898 (40·0%) responded to the baseline postal survey in 1998 and 18 468 (71·3%) had data on loneliness or social isolation and were linked to national health registries, contributing 180 650 person-years (figure 1). The mean age of participants was 42·5 years (SD 11·4); 11 367 (61·5%) were women and 7101 (38·5%) were men.

Among participants with available data, 4466 (24·4%) of 18 296 were classified as being lonely and 1776 (9·7%) of 18 376 as socially isolated. During a median follow-up of 10·0 years (IQR 10·0–10·1) we identified 814 (4·4%) participants with a hospital-treated infection and 680 (3·7%) all-cause deaths. Loneliness was associated with a higher risk of infection in the age-adjusted and sex-adjusted (HR 1·38 [95% CI 1·19–1·61]) and multivariable-adjusted (HR 1·32 [1·06–1·64]) models (appendix p 50). We found no association between social isolation and risk of hospital admission due to infection (age-adjusted and sex-adjusted HR 1·08 [95% CI 0·87–1·35]). We also found no strong evidence of reverse causation, although the associations were less precise due to smaller sample size. In a subgroup of participants who were not lonely at baseline, multivariable-adjusted HR of becoming lonely at follow-up was 1·10 (95% CI 0·66–1·75) for individuals with a history of hospital-treated infection at or before baseline compared with those without such a history. In a subgroup of participants who were not socially isolated at baseline, the corresponding multivariable-adjusted HR of becoming socially isolated at follow-up was 0·99 (95% CI 0·51–1·74; appendix p 50).

## Discussion

In this analysis of data from the UK Biobank and the nationwide Finnish HeSSup study, we found that lonely individuals are at a moderately increased risk of severe infectious diseases requiring hospital treatment. Excess risk of hospital-treated infections among people with loneliness was observed across participants differing in age, sex, socioeconomic background, chronic illnesses, and depressed mood in the UK Biobank, and this finding was replicated in the independent HeSSup study population. The increased risk was not explained by differences in socioeconomic and lifestyle risk factors, prevalent chronic conditions, and depressed mood between individuals who were lonely and those who were not. Analyses of repeated data on loneliness from the HeSSup study suggested that reverse causation was an unlikely explanation for this finding. We found no association between social isolation and severe infections in either cohort.

Our findings support the idea that loneliness and isolation only partly overlap. By referring to a discrepancy between desired and actual social relationships, loneliness represents a source of psychological stress.^6^, ^7^ However, social isolation refers to a lack of contacts within a person's social network or community and does not necessarily mean loneliness, as privacy can also be a person's preferred choice. This distinction might partly explain differences in associations between risk of severe infectious diseases and loneliness and social isolation. Chronic psychological stress is associated with systemic inflammation, immunosuppression, and reduced host resistance to infectious diseases.^7^, ^19^, ^23^, ^24^, ^25^, ^26^, ^27^ A meta-analysis of epidemiological evidence linked loneliness with stress-related inflammatory markers, such as interleukin-6, whereas no such association was observed for social isolation.^28^ Further longitudinal research is needed to examine stress and other potential mediators for the association between loneliness and hospital-treated infections.

On average, hospital admissions for infections occur at older ages and those for mental and behavioural problems at younger ages.^29^ This temporal order raises the hypothesis that loneliness and associated mental health problems might set in motion a cascade of physical diseases that increase susceptibility to severe infections. In this study, about 20% of the association between loneliness and infections was attributable to depressed mood. Loneliness was also prospectively associated with an increased risk of hospital-treated mental and behavioural disorders and, to a lesser extent, diseases of the respiratory, endocrine, nervous, and musculoskeletal systems.

Severe diseases (such as infections requiring hospital treatment) might potentially reduce social activities and elicit feelings of loneliness. This temporal order can introduce reverse causation, in which the observed association between loneliness and infectious diseases is attributable to the effect of the disease on psychosocial factors rather than vice versa. With repeated data on loneliness and social isolation in the HeSSup study, we were able to examine whether hospital-treated infections were associated with an increase in loneliness, but did not find any strong evidence to support this.

The present study has several strengths. With more than 900 hospital-treated infectious diseases and almost 500 000 participants from two separate cohorts, the present study provides a comprehensive examination of the associations of loneliness and social isolation with severe infectious diseases. The information on infectious diseases was obtained from national health registries that had comprehensive recording systems for hospital admissions. As such, the follow-up was almost complete and independent of active participation in the studies. Additionally, convergent findings from the two independent cohort studies in two different settings (the UK and Finland) with different baseline timings (2006–10 and 1998), and with different demographic characteristics (mean age 56·5 years in the UK Biobank *vs* 42·5 years in the HeSSup study) strengthens the external validity and supports the generalisability of these findings. In support of the robustness of our findings, repeating the analyses using a simpler single-item measure of loneliness produced similar results to those of the combined measure used in the main analyses.

This study had several limitations. Because we used non-randomised observational data, we cannot draw conclusions about causality or exclude residual confounding by unmeasured or imprecisely measured covariates. The response rate from the UK Biobank was only 5·5%, which might have affected the incidence of infectious diseases, although previous analyses have suggested a close agreement between findings from the UK Biobank and representative UK samples for risk factor–disease associations.^30^ The proportion of missing baseline data among the respondents was less than 10% in the UK Biobank study and 29% in the HeSSup study. However, in both studies, we avoided many dropouts during follow-up, which can introduce a more substantial bias than missing baseline data. With our outcome of interest defined as hospital admissions with a primary diagnosis of infection, our study shares the limitations of other studies using electronic health records of hospital admissions.

In conclusion, our findings suggest that lonely people are at a moderately increased risk of severe hospital-treated infections. Further research is needed on the subtypes of infectious diseases, including chronicity, pathogens, and disease invasiveness and severity. Interventional studies are required before policy recommendations can be advanced.

## Data sharing

We have provided derived variables for the administrators of the UK Biobank and Finnish Health and Social Support (HeSSup) studies. Researchers registered with UK Biobank can apply for access to the database by completing an application, which includes a summary of the research plan, data fields required, any new data or variables that will be generated, and payment to cover the incremental costs of servicing an application (https://www.ukbiobank.ac.uk/enable-your-research/applyfor-access). In the HeSSup study, pseudonymised questionnaire data as used in this study can be shared by request to the investigators (sakari.suominen@utu.fi). Linked health records require separate permission from the National Institute of Health and Welfare and Statistics Finland. The payment covers the incremental costs of servicing an application. We provide all the codes for replicating our results in the appendix (pp 11–45) so that any researcher with access to the UK Biobank and HeSSup studies can replicate our findings from the raw data.

## Declaration of interests

We declare no competing interests.
