# Supplementary material for: Association of social isolation and loneliness with risk of incident hospital-treated infections: an analysis of data from the UK Biobank and Finnish Health and Social Support studies
Source: Lancet Public Health. 2023 Jan 17;8(2):e109–18. doi: 10.1016/S2468-2667(22)00253-5 (PMC9879771; doi:10.1016/S2468-2667(22)00253-5)
Supplement: Supplementary appendix [file mmc1.pdf]

THE LANCET
Public Health

Supplementary appendix

This appendix formed part of the original submission and has been peer reviewed. We post it as supplied by the authors.

Supplement to: Elovainio M, Komulainen K, Sipilä PN, et al. Association of social isolation and loneliness with risk of incident hospital-treated infections: an analysis of data from the UK Biobank and Finnish Health and Social Support studies. *Lancet Public Health* 2023; published online Jan 17. https://doi.org/10.1016/S2468-2667(22)00253-5.

APPENDIX

Contents

Additional information about exposures and covariates	2
Additional information about outcome measures	5
Additional information about statistical methods	10
Additional results	45
References	51

Additional information about exposures and covariates

Loneliness refers to the discrepancy between the desired and actual quality/quantity of social relationships that a person experiences, while social isolation is denoted by a lack of contacts within a social network or community.[1,2] By referring to a discrepancy between the desired and actual social relationships, loneliness represents a source of psychological stress.[3] Social isolation, in turn, refers to a lack of contacts within a person's social network or community and does not necessarily mean loneliness as privacy can be a person's preferred choice rather than an unwanted stressor.[4]

In UK Biobank, reference for loneliness and social isolation questions is [5] and we used the same categorisations as in the following studies. [6-8] Reference for the UCLA scale is [9]. Reference for the loneliness and social isolation questions is [10]. In a sensitivity analysis, we used a single-item measure of loneliness. This measure was comparable to the UCLA scale that is the most common way to measure loneliness in population surveys.[9] Previous studies have shown that simple single-item measures correlate highly with longer and more established measures, the correlations with the three-item UCLA measure being around 0.88.[11] Thus, individuals rating themselves as lonely on the UCLA items are also likely to rate themselves as lonely on direct measures.[12]

In agreement with previous studies,[6-8,11] we used binary variables to assess loneliness and social isolation. While this facilitates comparability across investigations, continuous variables or weighted scores capture a greater variability in loneliness and social isolation. Thus, use of binary variables could have strengthened or weakened associations if the uncovered variability affected disease risk.

The covariates used in the present study have been used in multivariable-adjusted models in previous studies.[6,13-15] These included demographic characteristics, which may act as confounders, [2] and socioeconomic factors, health behaviours, long-term illnesses, systemic inflammation and depressed mood which may act as confounders or mediators or both (**figure S1**). Several studies suggest demographic characteristics, such as age, sex, and ethnicity, may act as confounders for the associations between loneliness, social isolation and injections.[16-20] References suggesting that the other covariates may act as confounders or mediators or both include [16-19,21]. References for specific covariates include the following: Townsend index[22], Patient Health Questionnaire[23], the Beck Depression Inventory,[24] Metabolic Equivalent of Tasks,[25] (selected threshold).[25]

Physical conditions at baseline that according to the US Centers for Disease Prevention and Control (CDC, 2017) increase the risk of infectious diseases: any cancers (ICD-10 codes: C00 -C96), chronic kidney disease (ICD-10 code: N18), chronic lung diseases (ICD-10 codes: J44, J43, J45, J46, J84, E84, I27), dementia and related neurological conditions (ICD-10 codes: F00, F01, F02, F03, G30, G31), diabetes (type 1 or type 2) (ICD-10 codes: E10, E11, E12, E13, E14), Down syndrome (ICD-10 code: Q90), heart conditions and hypertension (ICD-10 codes: I50, I20, I21, I22, I23, I24, I25, I42, I10, I11, I12, I13, I14, I15), HIV infection (ICD-10 code: Z21, B20 -B24), primary immune deficiency diseases (ICD-10 codes:D80, D81, D82, D83, D84, D85, D86, D87, D88, D89), liver disease (ICD-10 codes: K70, K71, K72, K73, K74, K75, K76, K77), sickle cell disease or thalassemia (ICD-10 codes: D56, D57), stroke (ICD-10 codes: I60, I61, I63, I64) and substance use disorder (ICD-10 codes: F10, F11, F12, F13, F14, F15, F16, F17, F18, F19).

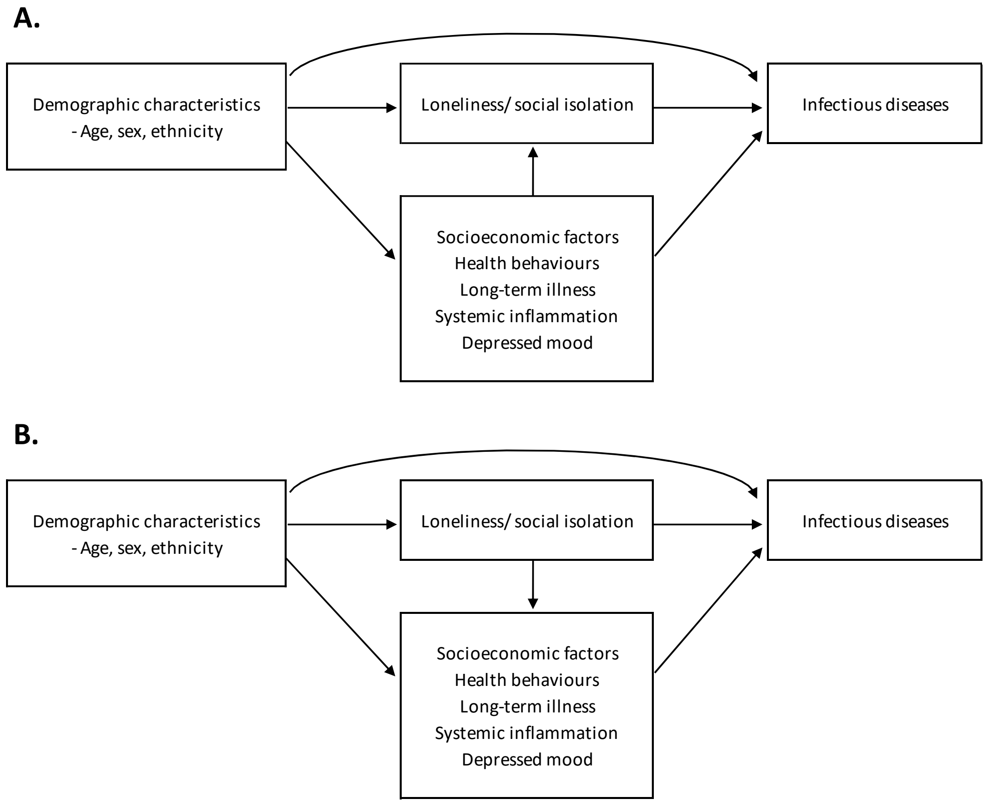

Figure S1. Two alternative directed acyclic graphs (DAGs) for exposures, covariates and outcomes (A: all covariates as confounders, B: covariates as confounders and mediators)

Table S1 shows correlation matrix for covariates.

Table S1. Correlation matrix for covariates in the UK Biobank cohort (N = 456,905 – 420,856 depending on the pair of covariates)

Age	Sex	Ethnicity	Education	Townsend index	Alcohol consumption	BMI	Smoking status	Physical activity	Long-standing illness	Depressive mood
0.02****										
0.12****	0.00									
-0.22****	0.02****	-0.04****								
-0.09****	0.01****	-0.19****	-0.10****							
0.05****	0.15****	0.12****	0.14****	-0.11****						
0.05****	0.08****	-0.02****	-0.13****	0.09****	-0.11****					
-0.08****	0.06****	-0.01****	-0.08****	0.16****	0.01****	-0.02****				
0.05****	0.02****	-0.01****	-0.08****	0.05****	-0.01****	-0.07****	0.02****			
0.24****	0.05****	0.00*	-0.12****	0.07****	-0.05****	0.22****	0.01****	-0.01****		
-0.12****	-0.06****	-0.07****	-0.04****	0.12****	-0.06****	0.05****	0.08****	-0.01****	0.08****	
0.06****	-0.05****	-0.01****	-0.10****	0.07****	-0.07****	0.33****	0.07****	-0.03****	0.13****	0.04****

**** p<0.001

Additional information about outcome measures

We excluded all participants with hospital episodes before the baseline from the analyses and used the first hospital treated the first hospital-treated infection as the main outcome. This approach corresponds to those used for other hospital-treated health outcomes in the present study and other studies.[6-8,11,26] The most common diagnoses for the 42,517 excluded participants with hospital-treated infection before or at baseline were ICD-10 N39.0 (Urinary tract infection, N = 8233), J18 (Pneumonia, N = 5278), L03 (Cellulitis and acute lymphangitis, N=5238), J22 (Unspecified acute lower respiratory infection, N =5173), B96.8 (Other specified bacterial agents as the cause of diseases classified elsewhere, N=4393) and B95 (Streptococcus, Staphylococcus, and Enterococcus as the cause of diseases classified elsewhere, N=3037). Electronic health records include cause and date of hospitalisation and their coverage (all hospital types, including private hospitals, and records cover emergencies) reflects the comprehensive nature of the public health-care system in the UK and Finland. Electronic health records did not include primary care visits or long-term care, such as provided in nursing homes.

With our primary endpoint defined as hospitalised cases with primary diagnosis of infections, our study shares the limitations of other studies using electronic health records from hospitalisations. The cases included only infections requiring hospitalisation whereas participants who were not infected plus those with no undiagnosed conditions or infections treated in primary care belonged to the non-case group. This means that our results are likely to reflect associations with serious infections and cannot be generalised to infectious diseases in general.

Broad ICD-10 disease categories for infectious diseases were as follows:[27]

A08.0	Rotaviral enteritis
A08.1	Acute gastroenteropathy due to Norwalk agent
A08.2	Adenoviral enteritis
A08.3	Other viral enteritis
A08.4	Viral intestinal infection, unspecified
A60*	Anogenital herpesviral [herpes simplex] infection
A63.0	Anogenital (venereal) warts
A80*	Acute poliomyelitis
A81.1	Subacute sclerosing panencephalitis
A81.2	Progressive multifocal leukoencephalopathy
A83*	Mosquito-borne viral encephalitis
A84*	Tick-borne viral encephalitis
A85*	Other viral encephalitis, not elsewhere classified
A86*	Unspecified viral encephalitis
A87*	Viral meningitis
A88*	Other viral infections of central nervous system, not elsewhere classified
A89*	Unspecified viral infection of central nervous system
A90*	Dengue fever [classical dengue]
A91*	Dengue haemorrhagic fever
A92	Other mosquito-borne viral fevers
A92.0	Chikungunya virus disease
A92.1	O'nyong-nyong fever

A92.2	Venezuelan equine fever
A92.3	West Nile virus infection
A92.4	Rift Valley fever
A92.8	Other specified mosquito-borne viral fevers
A92.9	Mosquito-borne viral fever, unspecified
A93	Other arthropod-borne viral fevers, not elsewhere classified
A93.0	Oropouche virus disease
A93.1	Sandfly fever
A93.2	Colorado tick fever
A93.8	Other specified arthropod-borne viral fevers
A94*	Unspecified arthropod-borne viral fever
A95*	Yellow fever
A96*	Arenaviral haemorrhagic fever
A97*	Dengue
A98	Other viral haemorrhagic fevers, not elsewhere classified
A98.0	Crimean-Congo haemorrhagic fever
A98.1	Omsk haemorrhagic fever
A98.2	Kyasanur Forest disease
A98.3	Marburg virus disease
A98.4	Ebola virus disease
A98.5	Haemorrhagic fever with renal syndrome
A98.8	Other specified viral haemorrhagic fevers
A99*	Unspecified viral haemorrhagic fever
B00	Herpesviral [herpes simplex] infections
B00.0	Eczema herpeticum
B00.1	Herpesviral vesicular dermatitis
B00.2	Herpesviral gingivostomatitis and pharyngotonsillitis
B00.3	Herpesviral meningitis
B00.4	Herpesviral encephalitis
B00.5	Herpesviral ocular disease
B00.7	Disseminated herpesviral disease
B00.8	Other forms of herpesviral infection
B00.9	Herpesviral infection, unspecified
B01	Varicella [chickenpox]
B01.0	Varicella meningitis
B01.1	Varicella encephalitis
B01.2	Varicella pneumonia
B01.8	Varicella with other complications
B01.9	Varicella without complication
B02	Zoster [herpes zoster]
B02.0	Zoster encephalitis
B02.1	Zoster meningitis
B02.2	Zoster with other nervous system involvement

B02.3	Zoster ocular disease
B02.7	Disseminated zoster
B02.8	Zoster with other complications
B02.9	Zoster without complication
B04*	Monkeypox
B05	Measles
B05.0	Measles complicated by encephalitis
B05.1	Measles complicated by meningitis
B05.2	Measles complicated by pneumonia
B05.3	Measles complicated by otitis media
B05.4	Measles with intestinal complications
B05.8	Measles with other complications
B05.9	Measles without complication
B06	Rubella [German measles]
B06.0	Rubella with neurological complications
B06.8	Rubella with other complications
B06.9	Rubella without complication
B07*	Viral warts
B08.0	Other orthopoxvirus infections
B08.1	Molluscum contagiosum
B08.2	Exanthema subitum [sixth disease]
B08.3	Erythema infectiosum [fifth disease]
B08.4	Enteroviral vesicular stomatitis with exanthem
B08.5	Enteroviral vesicular pharyngitis
B08.8	Other specified viral infections characterized by skin and mucous membrane lesions
B09*	Unspecified viral infection characterized by skin and mucous membrane lesions
B15	Acute hepatitis A
B15.0	Hepatitis A with hepatic coma
B15.9	Hepatitis A without hepatic coma
B16	Acute hepatitis B
B16.0	Acute hepatitis B with delta-agent (coinfection) with hepatic coma
B16.1	Acute hepatitis B with delta-agent (coinfection) without hepatic coma
B16.2	Acute hepatitis B without delta-agent with hepatic coma
B16.9	Acute hepatitis B without delta-agent and without hepatic coma
B17*	Other acute viral hepatitis
B18*	Chronic viral hepatitis
B19	Unspecified viral hepatitis
B19.0	Unspecified viral hepatitis with hepatic coma
B19.9	Unspecified viral hepatitis without hepatic coma
B20*	Human immunodeficiency virus [HIV] disease resulting in infectious and parasitic diseases
B21*	Human immunodeficiency virus [HIV] disease resulting in malignant neoplasms
B21.0	HIV disease resulting in Kaposi sarcoma
B22	Human immunodeficiency virus [HIV] disease resulting in other specified diseases

B22.0	HIV disease resulting in encephalopathy
B22.1	HIV disease resulting in lymphoid interstitial pneumonitis
B22.2	HIV disease resulting in wasting syndrome
B22.7	HIV disease resulting in multiple diseases classified elsewhere
B23*	Human immunodeficiency virus [HIV] disease resulting in other conditions
B24*	Unspecified human immunodeficiency virus [HIV] disease
B25*	Cytomegaloviral disease
B26	Mumps
B26.0	Mumps orchitis
B26.1	Mumps meningitis
B26.2	Mumps encephalitis
B26.3	Mumps pancreatitis
B26.8	Mumps with other complications
B26.9	Mumps without complication
B27	Infectious mononucleosis
B27.0	Gammaherpesviral mononucleosis
B27.1	Cytomegaloviral mononucleosis
B27.9	Infectious mononucleosis, unspecified
B30*	Viral conjunctivitis
B33	Other viral diseases, not elsewhere classified
B33.0	Epidemic myalgia
B33.1	Ross River disease
B33.2	Viral carditis
B33.3	Retrovirus infections, not elsewhere classified
B33.4	Hantavirus (cardio-)pulmonary syndrome [HPS] [HCPS]
B33.8	Other specified viral diseases
B34	Viral infection of unspecified site
B34.0	Adenovirus infection, unspecified site
B34.1	Enterovirus infection, unspecified site
B34.2	Coronavirus infection, unspecified site
B34.3	Parvovirus infection, unspecified site
B34.4	Papovavirus infection, unspecified site
B34.8	Other viral infections of unspecified site
B34.9	Viral infection, unspecified
B97	Viral agents as the cause of diseases classified to other chapters
B97.0	Adenovirus as the cause of diseases classified to other chapters
B97.1	Enterovirus as the cause of diseases classified to other chapters
B97.2	Coronavirus as the cause of diseases classified to other chapters
B97.3	Retrovirus as the cause of diseases classified to other chapters
B97.4	Respiratory syncytial virus as the cause of diseases classified to other chapters
B97.5	Reovirus as the cause of diseases classified to other chapters
B97.6	Parvovirus as the cause of diseases classified to other chapters
B97.7	Papillomavirus as the cause of diseases classified to other chapters

B97.8	Other viral agents as the cause of diseases classified to other chapters
C46	Kaposi sarcoma
G02.0	Meningitis in viral diseases classified elsewhere
G04.1	Tropical spastic paraplegia
G05.1	Encephalitis, myelitis and encephalomyelitis in viral diseases classified elsewhere
H19.1	Herpesviral keratitis and keratoconjunctivitis
H62.1	Otitis externa in viral diseases classified elsewhere
H67.1	Otitis media in viral diseases classified elsewhere
I41.1	Myocarditis in viral diseases classified elsewhere
J09*	Influenza due to certain identified influenza virus
J10*	Influenza due to other identified influenza virus
J11*	Influenza, virus not identified
J12*	Viral pneumonia, not elsewhere classified
J17.1	Pneumonia in viral diseases classified elsewhere
J20.3	Acute bronchitis due to coxsackievirus
J20.4	Acute bronchitis due to parainfluenza virus
J20.5	Acute bronchitis due to respiratory syncytial virus
J20.6	Acute bronchitis due to rhinovirus
J20.7	Acute bronchitis due to echovirus
J21*	Acute bronchiolitis
J21.0	Acute bronchiolitis due to respiratory syncytial virus
J21.1	Acute bronchiolitis due to human metapneumovirus
J21.8	Acute bronchiolitis due to other specified organisms
J21.9	Acute bronchiolitis, unspecified
M01.4	Rubella arthritis
M01.5	Arthritis in other viral diseases classified elsewhere
O98.4	Viral hepatitis complicating pregnancy, childbirth and the puerperium
O98.5	Other viral diseases complicating pregnancy, childbirth and the puerperium
O98.7	Human immunodeficiency virus [HIV] disease complicating pregnancy, childbirth and the puerperium
P23.0	Congenital pneumonia due to viral agent
P35.0	Congenital rubella syndrome
P35.1	Congenital cytomegalovirus infection
P35.2	Congenital herpesviral [herpes simplex] infection
P35.3	Congenital viral hepatitis
Z21	Asymptomatic human immunodeficiency virus [HIV] infection status

ICD-10-codes for contagious infections were as follows: influenza (J09-J11), gastrointestinal infections (A00-A09), or may be activated through biological process caused by psychological stress, including pneumonia (J12-J18), urinary tract infections (N10, N13.6, N15.1, N30.0, N39.0) or skin and subcutaneous tissue infections (A46, L00-L08).

Disease categories in an outcome-wide analysis were as follows: neoplasms (ICD-10 codes C00-D48), diseases of the blood (D50-D89), endocrine diseases (E00-E99), mental and behavioural disorders (F00-F99), diseases of the nervous system (G00-G99), diseases of the eye and ear (H00-H95), diseases of the circulatory system (I00-I99), diseases of the respiratory system (J00-J99), diseases of the digestive system (K00-K93), diseases of the skin

(L00-L99), diseases of the musculoskeletal system (M00-M99), and diseases of the genitourinary system (N00-N99).

Additional information about statistical methods

We tested proportional hazards assumption formally with interaction terms of log(time) with loneliness and social isolation. There was a significant interaction with loneliness ($p<0.001$) but not with isolation ($p = 0.08$). In further analysis of the extent to which hazard ratios from a short follow-up (years 0-3) and long follow-up (from year 3 onwards) differed for loneliness, we found that the difference in hazard ratios was small (HR =1.42, 95% CI 1.35-1.51 versus 1.36, 95% CI 1.31-1.42) between the two periods of follow-up, a finding also confirmed by the log-log plots (**figure S2**). That this small difference reached conventional levels of statistical significance was due to the large sample size.

For main analysis, there would have been other analytic options, such using count outcome and negative binomial regression or recurrent event survival analyses, which however would not allow outcome-wide comparisons. Further studies using alternative analytic approaches could shed light into the extent to which loneliness and social isolation are linked to recurrent infections.

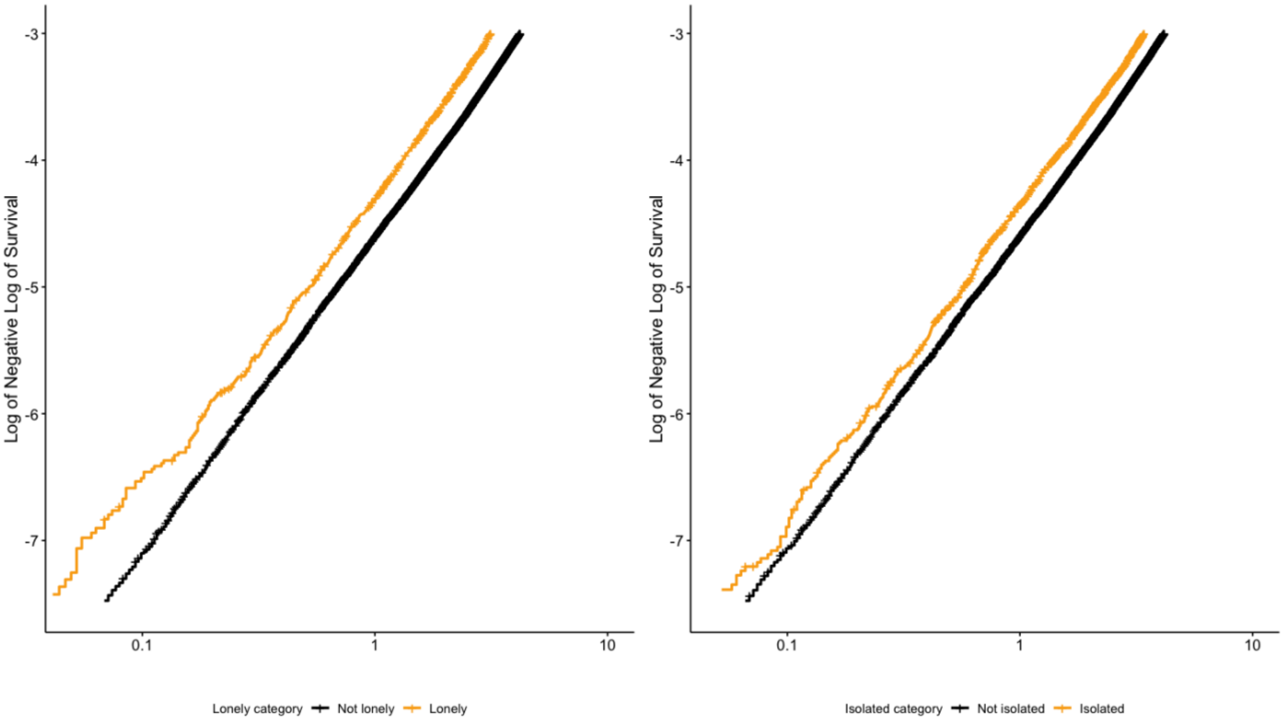

Figure S2. **Log-log plot for the associations of loneliness and social isolation with infectious diseases.**

The percentage of excess risk attributable to covariates (PERM) was calculated using the following formula:[28]

PERM = [Hazard ratio (age and sex adjusted) – hazard ratio (age, sex and covariate adjusted)]/ [Hazard ratio (age and sex adjusted) - 1] x 100."

To examine whether the associations of loneliness and social isolation with infectious diseases involve specific types of infections, we tested their associations with infections that are mainly contagious. To compare the associations with infectious diseases to those with other disease categories, we performed an outcome-wide analysis examining the associations of loneliness and social isolation with other broad disease categories, including separate analyses for neoplasms, diseases of the blood, endocrine diseases, mental and behavioural disorders, diseases of the nervous system, diseases of the eye and ear, diseases of the circulatory system, diseases of the respiratory system, diseases of the digestive system, diseases of the skin, diseases of the musculoskeletal system, and diseases of the genitourinary system. Effect estimates were adjusted for age, sex, education, ethnicity, deprivation index, alcohol consumption, smoking status, BMI, physical activity, long-term illness, CRP, and depressed mood.

The statistical code used was as follows:

R-code for the analyses

```
####################################################################################
###-------------    Inflammation analyses UKB   ------------------------####
####################################################################################
da4 is the primary data
da4 <- da4 %>%  rowwise %>%
  mutate(isolation = sum(livingalone,nofriends,noactivities)) %>%
  mutate(lonely=sum(noconfide,loneliness)) %>%
  mutate(isolated = case_when(
    isolation== 0 ~ 0,
    isolation== 1 ~ 0,
    isolation== 2 ~ 1,
    isolation== 3 ~ 1)) %>%
  mutate(lonelyd = case_when(
    lonely== 0 ~ 0,
    lonely== 1 ~ 0,
    lonely== 2 ~ 1)) %>% ungroup()
da4$mod<-dplyr::recode(da4$modphysical, `0` = 0, `1` = 0, `2` = 0,`3` = 0, `4` = 0,`5` = 1, `6` = 1, `7` = 1)
da4$vig<-dplyr::recode(da4$vigphysical, `0` = 0, `1` = 0, `2` = 0,`3` = 0, `4` = 0,`5` = 1, `6` = 1, `7` = 1 )
da4 <- da4 %>%
  mutate(modc=rec(modphysical, rec = "min:4=1; 5:7=2"), useNA = "always") %>%
  mutate(vigc=rec(vigphysical, rec = "min:4=1; 5:7=2"), useNA = "always")
da4 <- da4 %>%
  mutate(physical= case_when(mod==1 & vig==1 ~ "1", # both
                             xor(mod==1, vig==1) ~ "1", # one
                             !mod==1 & !vig==1 ~ "0")) # neither
da4$inco<-dplyr::recode(da4$hhincome, `1` = 1, `2` = 1, `3` = 2, `4` = 2,`5` = 2)
da4 <- da4%>% mutate(dep_l2wb=if_else((dep_l2w==4 | dep_l2w==3), 1, 0)) %>%
mutate(crp_over= if_else(crp_1>3, 1,0)) %>% mutate(dead=ifelse(is.na(date_of_death), 0, 1))
da4 <- da4%>% mutate(crp_class=rec(crp_1, rec="min:2.999999=1; 3:9.999999=2; 10:max=3"), useNA =
"always")
da4 <- da4%>% mutate(tow_q = ntile(deprivation_index , 3))
# Replacing missing inflammation with 0
# replace0 <- function(x) {
#   if_else(condition = is.na(x),
#           true = 0,
#           false = as.numeric(x))
# }
da4$anyanyinf <- replace0(da4$anyanyinf)
```

```
da4 <-  da4%>% mutate(
  cause4 = case_when(
    anyanyinf == 1 ~ 1, # "inflammation"
    is.na(date_of_death) & anyanyinf == 0 ~ 0, # "healthy"
    !is.na(date_of_death) & anyanyinf == 0 ~ 2)) # "died"
da4$anybactinf <- replace0(da4$anybactinf)
da4$anyviralinf <- replace0(da4$anyviralinf)
#---------------------------------------------------Dates-----------------------------------------------------#
da4$entrydateb <- da4$dateassessed
da4$exitdate_u <- da4$exitdate
da4 <- da4 %>% mutate(date_of_deathu= as_date(ifelse(date_of_death>lubridate::ymd(20180207), NA,
date_of_death)))
da4 <- da4 %>% mutate(exitdate_u= as_date(ifelse(exitdate <date_of_deathu, date_of_deathu, exitdate)))
da4 <- da4 %>% mutate(exitdate_u= as_date(ifelse(is.na(exitdate_u), exitdate, exitdate_u)))
da4 <- da4 %>% mutate(anyinf_date= if_else(is.na(ensianyinfpvm), exitdate_u,ensianyinfpvm))
da4 <- da4 %>% mutate(anyinf_date= if_else(is.na(anyinf_date),lubridate::ymd(20180207),anyinf_date))


da4 <- da4 %>% mutate(bactinf_date= if_else(is.na(ensibactinfpvm), exitdate_u,ensibactinfpvm))
da4 <- da4 %>% mutate(bactinf_date= if_else(is.na(bactinf_date),lubridate::ymd(20180207),bactinf_date))

da4 <- da4 %>% mutate(viralinf_date= if_else(is.na(ensiviralinfpvm), exitdate_u,ensiviralinfpvm))
da4 <- da4 %>% mutate(viralinf_date= if_else(is.na(viralinf_date),lubridate::ymd(20180207),viralinf_date))

library(magrittr)
library(survival)
da4$time_any <- as.numeric(difftime(da4$anyinf_date, da4$entrydateb, units = "weeks")/52)
 da4$time_bac <- as.numeric(difftime(da4$bactinf_date, da4$entrydateb, units = "weeks")/52)
 da4$time_vir <- as.numeric(difftime(da4$viralinf_date, da4$entrydateb, units = "weeks")/52)
da4 %>% select(anyinf_date) %>% filter(anyinf_date>lubridate::ymd(20180207))
# 459942-20905
da4a <- da4 %>% filter(time_any>0)
da4a <- da4a[!with(da4a,is.na(lonelyd) & is.na(isolated)),]
sum(is.na(da4a$lonelyd) & is.na(da4a$isolated))
456959+2983

#age groups for the table 1 (reviewer 1)
library(epikit)
da4a <- da4a %>%
  mutate(
    age_group = age_categories(
      age,
      breakers = c(0, 50, 60,99),
      ceiling = TRUE)) # 70 is ceiling, all above become NA
da5 <- da4a
frq(da5$bmi_who)
da5 <- within(da5, {
  sex <- factor(sex, labels = c("Female", "Male"))
  age_group <- factor(age_group, labels = c(" -49", "50 - 59", "60 - "))
  edugroup  <- factor(edugroup, labels = c("Lower", "Intermediate", "Higher"))
  anyill  <- factor(anyill, labels = c("No", "Yes"))
  physical  <- factor(physical, labels = c("Low", "High"))
  smoker<- factor(smoker , labels = c("No", "Yes"))
  alcweekly  <- factor(alcweekly, labels = c("Lower", "Higher"))
  dep_l2w  <- factor(dep_l2w, labels = c("Low", "Low-medium", "High_medium", "High"))
  dep_l2wb  <- factor(dep_l2wb, labels = c("Low", "High"))
  isolated<- factor(isolated, labels = c("No", "Yes"))
```

```
  lonelyd<- factor(lonelyd, labels = c("No", "Yes"))
  retired<- factor(retired, labels = c("No", "Yes"))
  crp_over<- factor(crp_over, labels = c("No", "Yes"))
  anyanyinf<- factor(anyanyinf, labels = c("No", "Yes"))
  tow_q<- factor(tow_q, labels = c("Lower", "Intermediate", "Higher"))
  white<- factor(white, labels = c("Other", "Caucasian"))
  bmi_who<- factor(bmi_who, labels = c("Normal", "Over_weight", "Obese"))
    })
da5$white <- factor(da5$white, levels=c("Caucasian", "Other"))
library(finalfit)
library(table1)
mydt <- da5 %>% select(
"Age_years" = age,
"Age_groups" =age_group,
"Sex" = sex,
"Race" = white,
"Education" = edugroup,
"Retired" = retired,
"Long_term_illness" = anyill,
"Physical_activity" = physical,
"Current_smoker" = smoker,
"Alcohol_consumption" = alcweekly,
"Depressive_mood" = dep_l2wb,
"Social_deprivation_index" = deprivation_index,
"Cognitive_performance" = iqscore,
"CRP_over_3_mg" = crp_over,
"Socially_isolated" =isolated,
"Feeling_lonely" = lonelyd,
"Any_infectious_disease" = anyanyinf,
"Follow_up_time_from_baseline_years"  = time_any,
"Townsend_index_tertile" =tow_q,
"BMI" =bmi,
"BMI_WHO"=bmi_who)

#New table for reviewer 1
table1(~ Sex+ Age_years + Age_groups+ Race
+Education+Townsend_index_tertile+Alcohol_consumption+Current_smoker+Physical_activity+BMI+BMI_WH
O+Depressive_mood+Long_term_illness+ CRP_over_3_mg +Socially_isolated + Feeling_lonely +
       Any_infectious_disease+ Follow_up_time_from_baseline_years, data=mydt, overall="Total",
      render.categorical="FREQ (PCTnoNA%)", caption = "<h3><b>Table 1, Characteristcs of the UK Biobank -
sample</b></h3>")
#original without missing
table1(~ Sex+ Age_years  + Education+Townsend_index_tertile+
Alcohol_consumption+Current_smoker+Physical_activity+Depressive_mood+Long_term_illness+
Socially_isolated + Feeling_lonely +
       Any_infectious_disease+ Follow_up_time_from_baseline_years, data=mydt, overall="Total",
render.missing=NULL,
      render.categorical="FREQ (PCTnoNA%)", caption = "<h3><b>Table 1, Characteristcs of the UK Biobank -
sample</b></h3>")

#-------------------- Checking proportional hazard assumption -------------------#

library(survminer)
glance(coxph(Surv(time_any, anyanyinf) ~ age+sex+lonelyd, data=da4a))
summary(coxph(Surv(time_any, anyanyinf) ~ age+sex+factor(depgroup) +lonelyd, data=da4a))
```

```
summary(coxph(Surv(time_any, anyanyinf) ~
age+sex+white+edugroup+deprivation_index+alcweekly+bmi+smoker+physical+anyill+dep_l2wb+crp_over+lo
nelyd, data=da4a))
res.cox <- coxph(Surv(time_any, anyanyinf) ~
age+sex+white+edugroup+deprivation_index+alcweekly+bmi+smoker+physical+anyill+dep_l2wb+crp_over+lo
nelyd+isolated, data=da4a)
res.cox <- coxph(Surv(time_any, anyanyinf) ~ age, data=da4a)
test.ph <- cox.zph(res.cox)
test.ph<- unclass(test.ph)
test.ph <- test.ph$table
test.ph <- as.data.frame(test.ph)
explanatory = c("Age_years", "Sex", "Ethnicity", "Education","Social_deprivation_index",
"Alcohol_consumption", "BMI","Current_smoker","Physical_activity", "Long_term_illness",
        "Depressive_mood", "CRP_over_3_mg", "Lonely", "Socially_isolated", "Global")

test.ph$variables <- explanatory
test.ph <- test.ph[, c(4, 1, 2, 3)]
tab_df(test.ph)
test.ph <- cox.zph(res.cox)
ggcoxzph(test.ph)

# da4a <- da4a %>% mutate(time_two= if_else(time_any<=6, 0, 1))
# frq(da4a$time_two)
# max(da4a$time_any)
res.cox2 <- coxph(Surv(time_any, anyanyinf) ~ age*log(time_any)+sex*log(time_any)+isolated*log(time_any),
data=da4a)
zcox1= coxph(Surv(short_time, short_anyinf) ~ age+sex+lonelyd, data=da4a)
tab_df(tidy(zcox1, exponentiate=T, conf.int=T))
zcox2 = coxph(Surv(long_time, anyanyinf) ~ age+sex+lonelyd, data=da5a)
tidy(zcox2, exponentiate=T, conf.int=T)


library(survival)
library(MASS)

456959/502506

fit<- survfit(Surv(time_any,anyanyinf)~ lonelyd, data=da4a)
prop1<-ggsurvplot(fit, data = da4a, fun = "cloglog", xlim = c(0.05, 10), ylim=c(-7.5, -3), palette=c("black",
"#FAAB18"),
         legend = "bottom", legend.title="Lonely category", legend.labs=c("Not lonely", "Lonely"), xlab =
"",ylab= "Log of Negative Log of Survival" )

fit2<- survfit(Surv(time_any,anyanyinf)~ isolated, data=da4a)
prop2<-ggsurvplot(fit2, data = da4a, fun = "cloglog", xlim = c(0.05, 10), ylim=c(-7.5, -3), palette=c("black",
"#FAAB18"),
         legend = "bottom", legend.title="Isolated category", legend.labs=c("Not isolated", "Isolated"), xlab =
"",ylab= "Log of Negative Log of Survival" )

splots <- list()
splots[[1]] <-prop1
splots[[2]] <-prop2


arrange_ggsurvplots(splots, ncol = 2, nrow = 1)
res <- arrange_ggsurvplots(splots, print = FALSE, title="Supplement figure . Log-log plot: log(follow-up) versus
log(-log(survival)) for outcomes with loneliness and isolation")
```

```
ggsave("/Users/melovain/Documents/kone/Akatemia20/UKBB_Infl/Kuvat/sfigure1.pdf", res)
dev.print(tiff , "/Users/melovain/Documents/kone/Akatemia20/UKBB_Infl/Kuvat/sfigure1", res = 100, height =
8, width = 12, units
        = "in", compression = "zip")


pfit <- pyears(Surv(time_any/365.25, anyanyinf) ~ 1, da4a)
glance(pfit)
sum(da4a$time_any, na.rm=T)
frq(da4a$anyanyinf)
mean(da4a$time_any)
#-----------------------Sample size calculations --------------------#
library(powerSurvEpi)
da4a$isos <- factor(da4a$isolated, labels=c("C", "E"))
frq(da4a$isos)
res1 <- ssizeCT(formula = Surv(time_any,anyanyinf)~ isos,
            dat = da4a,
            power = 0.8,
            k = 1,
            RR = 1.1,
            alpha = 0.05)
# the sample size
print(res1$ssize)

da4a$lones <- factor(da4a$lonelyd, labels=c("C", "E"))
frq(da4a$lones)
res2 <- ssizeCT(formula = Surv(time_any,anyanyinf)~ lones,
             dat = da4a,
             power = 0.8,
             k = 1,
             RR = 1.1,
             alpha = 0.05)
# the sample size
print(res2$ssize)

#-----------------------subgroup analyses ---------------------------#

library(epikit)
da4a <- da4a %>%
  mutate(
    age_group = age_categories(
      age,
      breakers = c(0, 50, 60,99),
      ceiling = TRUE)) # 70 is ceiling, all above become NA
# show table
hh0<-da4a %>%  do(tidy(coxph(Surv(time_any, anyanyinf) ~ age+sex+white+lonelyd, data=.), conf.int=TRUE,
exponentiate = TRUE))
hh0
hh1<-da4a %>% filter(!is.na(sex)) %>% group_by(sex) %>% do(tidy(coxph(Surv(time_any, anyanyinf) ~
age+white+ lonelyd, data=.), conf.int=TRUE,  exponentiate = TRUE))
hh1
hh2<-da4a %>% filter(!is.na(age_group)) %>% group_by(age_group) %>% do(tidy(coxph(Surv(time_any,
anyanyinf) ~ sex+white+lonelyd, data=.), conf.int=TRUE,  exponentiate = TRUE))
hh2
hh3<-da4a %>% filter(!is.na(edugroup)) %>% group_by(edugroup) %>% do(tidy(coxph(Surv(time_any,
anyanyinf) ~ sex+age+white+lonelyd, data=.), conf.int=TRUE,  exponentiate = TRUE))
hh3
```

```
hh4<-da4a %>% filter(!is.na(crp_class)) %>% group_by(crp_class) %>% do(tidy(coxph(Surv(time_any,
anyanyinf) ~ sex+age+white+lonelyd, data=.), conf.int=TRUE,  exponentiate = TRUE))
hh4
hh5<-da4a %>% filter(!is.na(anyill)) %>% group_by(anyill) %>% do(tidy(coxph(Surv(time_any, anyanyinf) ~
sex+age+white+lonelyd, data=.), conf.int=TRUE,  exponentiate = TRUE))
hh5
hh6<-da4a %>% filter(!is.na(dep_l2wb)) %>% group_by(dep_l2wb) %>% do(tidy(coxph(Surv(time_any,
anyanyinf) ~ sex+age+white+lonelyd, data=.), conf.int=TRUE,  exponentiate = TRUE))
hh6

hh0 <- hh0 %>% ungroup() %>% dplyr::select(term, estimate, conf.low, conf.high, p.value) %>%
filter(term=="lonelyd")
hh1 <- hh1 %>% ungroup() %>% dplyr::select(term, estimate, conf.low, conf.high, p.value) %>%
filter(term=="lonelyd")
hh2 <- hh2 %>% ungroup() %>% dplyr::select(term, estimate, conf.low, conf.high, p.value) %>%
filter(term=="lonelyd")
hh3 <- hh3 %>% ungroup() %>% dplyr::select(term, estimate, conf.low, conf.high, p.value) %>%
filter(term=="lonelyd")
hh4 <- hh4 %>% ungroup() %>% dplyr::select(term, estimate, conf.low, conf.high, p.value) %>%
filter(term=="lonelyd")
hh5 <- hh5 %>% ungroup() %>% dplyr::select(term, estimate, conf.low, conf.high, p.value) %>%
filter(term=="lonelyd")
hh6 <- hh6 %>% ungroup() %>% dplyr::select(term, estimate, conf.low, conf.high, p.value) %>%
filter(term=="lonelyd")

new.row0 <- data.frame(term="All", estimate= NA,conf.low =NA, conf.high =NA, p.value=
NA,stringsAsFactors=F)
new.row1 <- data.frame(term="Sex", estimate= NA,conf.low =NA, conf.high =NA, p.value= NA,
stringsAsFactors=F)
new.row2 <- data.frame(term="Age group", estimate= NA,conf.low =NA, conf.high =NA, p.value= NA,
stringsAsFactors=F)
new.row3 <- data.frame(term="Education", estimate= NA,conf.low =NA, conf.high =NA, p.value= NA,
stringsAsFactors=F)
new.row4 <- data.frame(term="CRP, mg/L", estimate= NA,conf.low =NA, conf.high =NA, p.value= NA,
stringsAsFactors=F)
new.row5<- data.frame(term="Long-term illness", estimate= NA,conf.low =NA, conf.high =NA, p.value= NA,
stringsAsFactors=F)
new.row6 <- data.frame(term="Depressive mood", estimate= NA,conf.low =NA, conf.high =NA, p.value= NA,
stringsAsFactors=F)
hh0 <- rbind(new.row0, hh0)
hh1 <- rbind(new.row1, hh1)
hh2 <- rbind(new.row2, hh2)
hh3 <- rbind(new.row3, hh3)
hh4 <- rbind(new.row4, hh4)
hh5 <- rbind(new.row5, hh5)
hh6 <- rbind(new.row6, hh6)

hhh <- rbind(hh0,hh1,hh2,hh3,hh4, hh5, hh6)

t0 <- da4a %>% do(m1=glance(coxph(Surv(time_any, anyanyinf) ~ lonelyd, data=da4a))) %>% unnest(m1)
t1 <- da4a %>% group_by(sex) %>% do(m1=glance(coxph(Surv(time_any, anyanyinf) ~ lonelyd, data=.))) %>%
unnest(m1)
t2 <- da4a %>% group_by(age_group) %>% do(m1=glance(coxph(Surv(time_any, anyanyinf) ~ lonelyd, data=.)))
%>% unnest(m1)
t3 <- da4a %>% group_by(edugroup) %>% do(m1=glance(coxph(Surv(time_any, anyanyinf) ~ lonelyd, data=.)))
%>% unnest(m1)
```

```
t4 <- da4a %>% group_by(crp_class) %>% do(m1=glance(coxph(Surv(time_any, anyanyinf) ~ lonelyd, data=.)))
%>% unnest(m1)
t5 <- da4a %>% group_by(anyill) %>% do(m1=glance(coxph(Surv(time_any, anyanyinf) ~ lonelyd, data=.))) %>%
unnest(m1)
t6 <- da4a %>% group_by(dep_l2wb) %>% do(m1=glance(coxph(Surv(time_any, anyanyinf) ~ lonelyd, data=.)))
%>% unnest(m1)

hhh[2,6]<-  paste(as.character(t0[1,1]))
hhh[4,6]<-  paste(as.character(t1[1,2]))
hhh[5,6]<-  paste(as.character(t1[2,2]))
hhh[7,6]<-  paste(as.character(t2[1,2]))
hhh[8,6]<-  paste(as.character(t2[2,2]))
hhh[9,6]<-  paste(as.character(t2[3,2]))
hhh[11,6]<- paste(as.character(t3[1,2]))
hhh[12,6]<- paste(as.character(t3[2,2]))
hhh[13,6]<- paste(as.character(t3[3,2]))
hhh[15,6]<- paste(as.character(t4[1,2]))
hhh[16,6]<- paste(as.character(t4[2,2]))
hhh[17,6]<- paste(as.character(t4[3,2]))
hhh[19,6]<- paste(as.character(t5[1,2]))
hhh[20,6]<- paste(as.character(t5[2,2]))
hhh[22,6]<- paste(as.character(t6[1,2]))
hhh[23,6]<- paste(as.character(t6[2,2]))
colnames(hhh)[6] <- c("Nncase")

hhh[2,7]<-  paste(as.character(t0[1,2]))
hhh[4,7]<-  paste(as.character(t1[1,3]))
hhh[5,7]<-  paste(as.character(t1[2,3]))
hhh[7,7]<-  paste(as.character(t2[1,3]))
hhh[8,7]<-  paste(as.character(t2[2,3]))
hhh[9,7]<-  paste(as.character(t2[3,3]))
hhh[11,7]<- paste(as.character(t3[1,3]))
hhh[12,7]<- paste(as.character(t3[2,3]))
hhh[13,7]<- paste(as.character(t3[3,3]))
hhh[15,7]<- paste(as.character(t4[1,3]))
hhh[16,7]<- paste(as.character(t4[2,3]))
hhh[17,7]<- paste(as.character(t4[3,3]))
hhh[19,7]<- paste(as.character(t5[1,3]))
hhh[20,7]<- paste(as.character(t5[2,3]))
hhh[22,7]<- paste(as.character(t6[1,3]))
hhh[23,7]<- paste(as.character(t6[2,3]))


hhh<-hhh %>% mutate(term = na_if(term, "lonelyd"))
hhh[1,1]<- ""
hhh[2,1]<- "All"
hhh[4,8]<- "Women"
hhh[5,8]<- "Men"
hhh[7,8]<- "40- 49 years"
hhh[8,8]<- "50 -59 years"
hhh[9,8]<- "60 - years"
hhh[11,8]<- "Lower"
hhh[12,8]<- "Intermediate"
hhh[13,8]<- "Higher"
hhh[15,8]<- " <3 mg/L"
hhh[16,8]<- "3 - <10 mg/L years"
hhh[17,8]<-"10 - mg/L"
```

```
hhh[19,8]<- "No"
hhh[20,8]<-"Yes"
hhh[22,8]<- "No"
hhh[23,8]<-"Yes"
hhhh<-hhh
library(extrafont)
library(sysfonts)
par(omi = c(0, 0, 0, 0)) # Outer margins back
par(family = "Times New Roman")
rowseq <- seq(nrow(hhhh),1)
par(mai=c(1,0,0,0))
plot(hhhh$estimate, rowseq, pch=15,cex = 1.5,
    xlim=c(1,1), ylim=c(0,25),
    xlab='', ylab='', yaxt='n', xaxt='n',
    bty='n', log= "x")
axis(1, round(log(seq(0.6,3,by=0.1))+1, 1), cex.axis=1.1, font=2)

segments(1,-1,1, 23, lty=3)
segments(hhhh$conf.low, rowseq, hhhh$conf.high, rowseq, cex=4, lwd=2)
text(0.1,(nrow(hhhh)+2.7), "A.", cex=1.2, font=2, pos=4)

text(0.5,(nrow(hhhh)+1), "Hazard ratio (95% confidence interval) for hospital treated infection", cex=1.2,
font=2, pos=4)
text(0.1,(nrow(hhhh)+1), "Study population", cex=1.2, font=2, pos=4)
t1h <- ifelse(!is.na(hhhh$term), hhhh$term, '')
text(0.1,rowseq, t1h, cex=1.2, pos=4, font=2)
t1 <- ifelse(!is.na(hhhh$V8), hhhh$V8, '')
text(0.1,rowseq, t1, cex=1.2, pos=4, font=1)

text(0.1,(nrow(hhhh)+2), "Loneliness", cex=1.2, font=2, pos=4)

t3b <- with(hhhh, paste(sprintf("%.2f",hhhh$estimate, 2),' (',sprintf("%.2f",conf.low, 2),' to
',sprintf("%.2f",conf.high, 2),')',sep=''))

t3 <- gsub("(NA to NA)","", t3b)
t3 <- gsub("NA ()", "", t3)
t3 <- gsub("\\()", "", t3)

text(2.2,rowseq, t3, cex=1.2, font =1, pos=4)

text(0.22,(nrow(hhhh)+1), "N(total)", cex=1.2, font=2, pos=4)
t6 <- with(hhhh, paste(Nncase))
t6 <- gsub("NA", "", t6)
text(0.22,rowseq, t6, cex=1.2, font =1, pos=4)

text(0.3,(nrow(hhhh)+1), "N(cases)", cex=1.2, font=2, pos=4)
t7 <- with(hhhh, paste(V7))
t7 <- gsub("NA", "", t7)
text(0.3,rowseq, t7, cex=1.2, font =1, pos=4)

segments(0.1, 23.2, 8.9,23.2   , lty=1, lwd=1.2)

# Interactions reviewer 3
modelsm <- list("age+factor(sex)*isolated", "sex+factor(age_group)*isolated",
"age+sex+factor(edugroup)*isolated","age+sex+factor(crp_class)*isolated","age+sex+factor(anyill)*isolated","
age+sex+factor(dep_l2wb)*isolated")list_out <- lapply(modelsm, function(i)
  round_df(tidy(coxph(as.formula(paste("Surv(time_any, anyanyinf) ~ ", i)), data = da4a))))
```

```
intd<- do.call(rbind, list_out) %>% filter(str_detect(term, ":"))
hhhh[5,9] <- intd[1,5]
hhhh[8,9] <- intd[2,5]
hhhh[9,9] <- intd[3,5]
hhhh[12,9] <- intd[4,5]
hhhh[13,9] <- intd[5,5]
hhhh[16,9] <- intd[6,5]
hhhh[17,9] <- intd[7,5]
hhhh[20,9] <- intd[8,5]
hhhh[23,9] <- intd[9,5]
hhhh[8,9] <- "0.70"
hhhh$p.value.1 <- as.character(hhhh$p.value.1)
text(4.4,(nrow(hhhh)+1), "p-value for interaction", cex=1.2, font=2, pos=4)
t9 <- with(hhhh, paste(p.value.1))
t9 <- gsub("NA", "", t9)
text(4.4,rowseq, t9, cex=1.2, font =1, pos=4)

dev.print(tiff , "/Users/melovain/Documents/kone/Akatemia20/UKBB_Infl/LPH_rew/Kuvat/Figure1a_b", res =
100, height = 10, width = 19, units
        = "in", compression = "zip")

#--------------------------------- Isolated -------------------------------------#

hh0<-da4a %>%  do(tidy(coxph(Surv(time_any, anyanyinf) ~ age+sex+white+isolated, data=.), conf.int=TRUE,
exponentiate = TRUE))
hh0
hh1<-da4a %>% filter(!is.na(sex)) %>% group_by(sex) %>% do(tidy(coxph(Surv(time_any, anyanyinf) ~
age+white+isolated, data=.), conf.int=TRUE,  exponentiate = TRUE))
hh1
hh2<-da4a %>% filter(!is.na(age_group)) %>% group_by(age_group) %>% do(tidy(coxph(Surv(time_any,
anyanyinf) ~ sex+white+isolated, data=.), conf.int=TRUE,  exponentiate = TRUE))
hh2
hh3<-da4a %>% filter(!is.na(edugroup)) %>% group_by(edugroup) %>% do(tidy(coxph(Surv(time_any,
anyanyinf) ~ age+sex+white+isolated, data=.), conf.int=TRUE,  exponentiate = TRUE))
hh3
hh4<-da4a %>% filter(!is.na(crp_class)) %>% group_by(crp_class) %>% do(tidy(coxph(Surv(time_any,
anyanyinf) ~  age+sex+white+isolated, data=.), conf.int=TRUE,  exponentiate = TRUE))
hh4
hh5<-da4a %>% filter(!is.na(anyill)) %>% group_by(anyill) %>% do(tidy(coxph(Surv(time_any, anyanyinf) ~
age+sex+ white+isolated, data=.), conf.int=TRUE,  exponentiate = TRUE))
hh5
hh6<-da4a %>% filter(!is.na(dep_l2wb)) %>% group_by(dep_l2wb) %>% do(tidy(coxph(Surv(time_any,
anyanyinf) ~  age+sex+white+isolated, data=.), conf.int=TRUE,  exponentiate = TRUE))
hh6

hh0 <- hh0 %>% ungroup() %>% dplyr::select(term, estimate, conf.low, conf.high, p.value) %>%
filter(term=="isolated")
hh1 <- hh1 %>% ungroup() %>% dplyr::select(term, estimate, conf.low, conf.high, p.value) %>%
filter(term=="isolated")
hh2 <- hh2 %>% ungroup() %>% dplyr::select(term, estimate, conf.low, conf.high, p.value) %>%
filter(term=="isolated")
hh3 <- hh3 %>% ungroup() %>% dplyr::select(term, estimate, conf.low, conf.high, p.value) %>%
filter(term=="isolated")
hh4 <- hh4 %>% ungroup() %>% dplyr::select(term, estimate, conf.low, conf.high, p.value) %>%
filter(term=="isolated")
hh5 <- hh5 %>% ungroup() %>% dplyr::select(term, estimate, conf.low, conf.high, p.value) %>%
filter(term=="isolated")
```

```
hh6 <- hh6 %>% ungroup() %>% dplyr::select(term, estimate, conf.low, conf.high, p.value) %>%
filter(term=="isolated")

new.row0 <- data.frame(term="All", estimate= NA,conf.low =NA, conf.high =NA, p.value=
NA,stringsAsFactors=F)
new.row1 <- data.frame(term="Sex", estimate= NA,conf.low =NA, conf.high =NA, p.value= NA,
stringsAsFactors=F)
new.row2 <- data.frame(term="Age group", estimate= NA,conf.low =NA, conf.high =NA, p.value= NA,
stringsAsFactors=F)
new.row3 <- data.frame(term="Education", estimate= NA,conf.low =NA, conf.high =NA, p.value= NA,
stringsAsFactors=F)
new.row4 <- data.frame(term="CRP, mg/L", estimate= NA,conf.low =NA, conf.high =NA, p.value= NA,
stringsAsFactors=F)
new.row5<- data.frame(term="Long-term illness", estimate= NA,conf.low =NA, conf.high =NA, p.value= NA,
stringsAsFactors=F)
new.row6 <- data.frame(term="Depressive mood", estimate= NA,conf.low =NA, conf.high =NA, p.value= NA,
stringsAsFactors=F)
hh0 <- rbind(new.row0, hh0)
hh1 <- rbind(new.row1, hh1)
hh2 <- rbind(new.row2, hh2)
hh3 <- rbind(new.row3, hh3)
hh4 <- rbind(new.row4, hh4)
hh5 <- rbind(new.row5, hh5)
hh6 <- rbind(new.row6, hh6)

hhh <- rbind(hh0,hh1,hh2,hh3,hh4, hh5, hh6)

t0 <- da4a %>% do(m1=glance(coxph(Surv(time_any, anyanyinf) ~ isolated, data=da4a))) %>% unnest(m1)
t1 <- da4a %>% group_by(sex) %>% do(m1=glance(coxph(Surv(time_any, anyanyinf) ~ isolated, data=.))) %>%
unnest(m1)
t2 <- da4a %>% group_by(age_group) %>% do(m1=glance(coxph(Surv(time_any, anyanyinf) ~ isolated,
data=.))) %>% unnest(m1)
t3 <- da4a %>% group_by(edugroup) %>% do(m1=glance(coxph(Surv(time_any, anyanyinf) ~ isolated, data=.)))
%>% unnest(m1)
t4 <- da4a %>% group_by(crp_class) %>% do(m1=glance(coxph(Surv(time_any, anyanyinf) ~ isolated, data=.)))
%>% unnest(m1)
t5 <- da4a %>% group_by(anyill) %>% do(m1=glance(coxph(Surv(time_any, anyanyinf) ~ isolated, data=.)))
%>% unnest(m1)
t6 <- da4a %>% group_by(dep_l2wb) %>% do(m1=glance(coxph(Surv(time_any, anyanyinf) ~ isolated, data=.)))
%>% unnest(m1)

hhh[2,6]<-  paste(as.character(t0[1,1]))
hhh[4,6]<-  paste(as.character(t1[1,2]))
hhh[5,6]<-  paste(as.character(t1[2,2]))
hhh[7,6]<-  paste(as.character(t2[1,2]))
hhh[8,6]<-  paste(as.character(t2[2,2]))
hhh[9,6]<-  paste(as.character(t2[3,2]))
hhh[11,6]<- paste(as.character(t3[1,2]))
hhh[12,6]<- paste(as.character(t3[2,2]))
hhh[13,6]<- paste(as.character(t3[3,2]))
hhh[15,6]<- paste(as.character(t4[1,2]))
hhh[16,6]<- paste(as.character(t4[2,2]))
hhh[17,6]<- paste(as.character(t4[3,2]))
hhh[19,6]<- paste(as.character(t5[1,2]))
hhh[20,6]<- paste(as.character(t5[2,2]))
hhh[22,6]<- paste(as.character(t6[1,2]))
hhh[23,6]<- paste(as.character(t6[2,2]))
```

```
colnames(hhh)[6] <- c("Nncase")

hhh[2,7]<-  paste(as.character(t0[1,2]))
hhh[4,7]<-  paste(as.character(t1[1,3]))
hhh[5,7]<-  paste(as.character(t1[2,3]))
hhh[7,7]<-  paste(as.character(t2[1,3]))
hhh[8,7]<-  paste(as.character(t2[2,3]))
hhh[9,7]<-  paste(as.character(t2[3,3]))
hhh[11,7]<- paste(as.character(t3[1,3]))
hhh[12,7]<- paste(as.character(t3[2,3]))
hhh[13,7]<- paste(as.character(t3[3,3]))
hhh[15,7]<- paste(as.character(t4[1,3]))
hhh[16,7]<- paste(as.character(t4[2,3]))
hhh[17,7]<- paste(as.character(t4[3,3]))
hhh[19,7]<- paste(as.character(t5[1,3]))
hhh[20,7]<- paste(as.character(t5[2,3]))
hhh[22,7]<- paste(as.character(t6[1,3]))
hhh[23,7]<- paste(as.character(t6[2,3]))

hhh<-hhh %>% mutate(term = na_if(term, "isolated"))
hhh[1,1]<- NA
hhh[2,1]<- "All"
hhh[4,8]<- "Women"
hhh[5,8]<- "Men"
hhh[7,8]<- "40 - 49 years"
hhh[8,8]<- "50 - 59 years"
hhh[9,8]<- "60 - years"
hhh[11,8]<- "Lower"
hhh[12,8]<- "Intermediate"
hhh[13,8]<- "Higher"
hhh[15,8]<- " <3 mg/L"
hhh[16,8]<- "3 - <10 mg/L years"
hhh[17,8]<-"10 - mg/L"
hhh[19,8]<- "No"
hhh[20,8]<-"Yes"
hhh[22,8]<- "No"
hhh[23,8]<-"Yes"
hhhh<-hhh

library(extrafont)
library(sysfonts)
par(omi = c(0, 0, 0, 0)) # Outer margins back
par(family = "Times New Roman")
rowseq <- seq(nrow(hhhh),1)
par(mai=c(1,0,0,0))
plot(hhhh$estimate, rowseq, pch=15,cex = 1.5,
    xlim=c(1,1), ylim=c(0,25),
    xlab='', ylab='', yaxt='n', xaxt='n',
    bty='n', log= "x")
axis(1, round(log(seq(0.6,3,by=0.1))+1, 1), cex.axis=1.1, font=2)

segments(1,-1,1, 23, lty=3)
segments(hhhh$conf.low, rowseq, hhhh$conf.high, rowseq, cex=4, lwd=2)
text(0.1,(nrow(hhhh)+2.7), "B.", cex=1.2, font=2, pos=4)
```

```
text(0.5,(nrow(hhhh)+1), "Hazard ratio (95% confidence interval) for hospital treated infection", cex=1.2,
font=2, pos=4)
text(0.1,(nrow(hhhh)+1), "Study population", cex=1.2, font=2, pos=4)
t1h <- ifelse(!is.na(hhhh$term), hhhh$term, '')
text(0.1,rowseq, t1h, cex=1.2, pos=4, font=2)
t1 <- ifelse(!is.na(hhhh$V8), hhhh$V8, '')
text(0.1,rowseq, t1, cex=1.2, pos=4, font=1)

text(0.1,(nrow(hhhh)+2), "Isolated", cex=1.2, font=2, pos=4)

t3b <- with(hhhh, paste(sprintf("%.2f",hhhh$estimate, 2),' (',sprintf("%.2f",conf.low, 2),' to
',sprintf("%.2f",conf.high, 2),')',sep=''))

t3 <- gsub("(NA to NA)","", t3b)
t3 <- gsub("NA ()", "", t3)
t3 <- gsub("\\()", "", t3)

text(2.2,rowseq, t3, cex=1.2, font =1, pos=4)

text(0.22,(nrow(hhhh)+1), "N(total)", cex=1.2, font=2, pos=4)
t6 <- with(hhhh, paste(Nncase))
t6 <- gsub("NA", "", t6)
text(0.22,rowseq, t6, cex=1.2, font =1, pos=4)

text(0.3,(nrow(hhhh)+1), "N(cases)", cex=1.2, font=2, pos=4)
t7 <- with(hhhh, paste(V7))
t7 <- gsub("NA", "", t7)
text(0.3,rowseq, t7, cex=1.2, font =1, pos=4)

segments(0.1, 23.2, 8.9,23.2   , lty=1, lwd=1.2)

#interaction fro reviewer 3
# Interactions reviewer 3
modelsm <- list("age+factor(sex)*isolated", "sex+factor(age_group)*isolated",
"age+sex+factor(edugroup)*isolated","age+sex+factor(crp_class)*isolated","age+sex+factor(anyill)*isolated","
age+sex+factor(dep_l2wb)*isolated")list_out <- lapply(modelsm, function(i)
  round_df(tidy(coxph(as.formula(paste("Surv(time_any, anyanyinf) ~ ", i)), data = da4a))))
intd<- do.call(rbind, list_out) %>% filter(str_detect(term, ":"))
hhhh[5,9] <- intd[1,5]
hhhh[8,9] <- intd[2,5]
hhhh[9,9] <- intd[3,5]
hhhh[12,9] <- intd[4,5]
hhhh[13,9] <- intd[5,5]
hhhh[16,9] <- intd[6,5]
hhhh[17,9] <- intd[7,5]
hhhh[20,9] <- intd[8,5]
hhhh[23,9] <- intd[9,5]
hhhh$p.value.1 <- as.character(hhhh$p.value.1)
hhhh[5,9] <- "0.20"
hhhh[13,9] <- "<0.001"
hhhh[16,9] <- "0.10"
hhhh[20,9] <- "<0.001"
text(4.4,(nrow(hhhh)+1), "p-value for interaction", cex=1.2, font=2, pos=4)
t9 <- with(hhhh, paste(p.value.1))
t9 <- gsub("NA", "", t9)
text(4.4,rowseq, t9, cex=1.2, font =1, pos=4)
# shaded_rowseq <- rowseq[rep(c(T,F),length(rowseq)/2)]
```

```
# rect(-10, shaded_rowseq-0.5, 12, shaded_rowseq+0.5, col="#00000025", border=NA)

dev.print(tiff , "/Users/melovain/Documents/kone/Akatemia20/UKBB_Infl/LPH_rew/Kuvat/Figure1b_b", res =
100, height = 10, width = 19, units
        = "in", compression = "zip")


da4a <- da4a %>% mutate(short_anyinf= if_else(anyanyinf==1 & time_any<=3, 1,0))
da4a <- da4a %>% mutate(short_time= if_else(time_any<=3, time_any, 3))
da5a<-da4a %>% filter(time_any>3) %>% mutate(long_time=time_any-3)

#---------------------------------- dropping those with risk diseases ---------------------------------------#
library(purrr)

 # "%nin%" <- function(x, y) {
 #   return( !(x %in% y) )
 # }

load("/Users/melovain/Documents/kone/Akatemia20/UKBB_Infl/all_other_diseases_f.Rdata")
da4d <- da4a %>% filter(id %nin% all_other_diseases_f$id)
da4d <- da4d %>% filter(time_any>0)

Cox_r1 = coxph( Surv(time_any, anyanyinf) ~ age+sex+lonelyd, data=da4d)
summary(Cox_r1)
Cox_r2 = coxph( Surv(time_any, anyanyinf) ~ age+sex+isolated, data=da4d)
summary(Cox_r2)
tidy(Cox_r1)

#------------------------------ analyses with only one item loneliness-measure ---- reviewer 4

frq(da4a$loneliness)

cox_oneitem1 = coxph(Surv(time_any, anyanyinf) ~ age+sex+white+loneliness, data=da4a)
summary(cox_oneitem1)

cox_oneitem2 = coxph(Surv(time_any, anyanyinf) ~
age+sex+white+edugroup+deprivation_index+alcweekly+bmi+smoker+physical+anyill+dep_l2w+crp_over+lon
eliness, data=da4a)
summary(cox_oneitem2)

#----------------- fully adjusted loneliness + isolation----------reviewer 4

cox_rew4 = coxph(Surv(time_any, anyanyinf) ~
age+sex+white+edugroup+deprivation_index+alcweekly+bmi+smoker+physical+anyill+dep_l2w+crp_over+isol
ated+lonelyd, data=da4a)
summary(cox_rew4)

#-------------------- correlation analyses in confonders---------reviewer 4
tab_df(corstars(da4a[c("age","sex","white",
"edugroup","deprivation_index","alcweekly","bmi","smoker","physical","anyill","dep_l2w","crp_over")]))
cor(da4a[c("age","sex","deprivation_index","alcweekly","bmi","anyill","dep_l2w","crp_over")])
tab_xtab(da4a$lonelyd,da4a$isolated, show.cell.prc = TRUE,show.row.prc = TRUE)

#----------------- all ICD-10 codes--------
library(pdftools)
rawlines <- pdf_text("/Users/melovain/Downloads/mmc1-7.pdf") %>%
  read_lines() %>%
```

```
  data.frame() %>%
  rename(rawline = 1)
tab_df(data.frame(rawlines[1162:3091,]))



#------------------------------------------- ANY INFLAMMATION ----------------------------------------------#
#Loneliness
Cox_1t = coxph(Surv(time_any, anyanyinf) ~ age+sex+factor(lonelyd), data=da4a)
summary(Cox_1t)
Cox_2t = coxph(Surv(time_any, anyanyinf) ~ age+sex+white +factor(lonelyd), data=da4a)
summary(Cox_2t)
Cox_3t = coxph(Surv(time_any, anyanyinf) ~ age+sex+white
+factor(edugroup)+deprivation_index+factor(lonelyd), data=da4a)
summary(Cox_3t)
Cox_4t = coxph(Surv(time_any, anyanyinf) ~ age+sex+white
+alcweekly+factor(bmi_who)+smoker+physical+factor(lonelyd), data=da4a)
summary(Cox_4t)
Cox_5t = coxph(Surv(time_any, anyanyinf) ~ age+sex+white +anyill+factor(lonelyd), data=da4a)
summary(Cox_5t)
Cox_6t = coxph(Surv(time_any, anyanyinf) ~ age+sex+white +dep_l2wb+factor(lonelyd), data=da4a)
summary(Cox_6t)
Cox_7t = coxph(Surv(time_any, anyanyinf) ~ age+sex+white +factor(crp_class)+factor(lonelyd), data=da4a)
summary(Cox_7t)
Cox_8t = coxph(Surv(time_any, anyanyinf) ~ age+sex+white
+factor(edugroup)+deprivation_index+alcweekly+factor(bmi_who)+smoker+physical+anyill+dep_l2w+factor(cr
p_class)+factor(lonelyd), data=da4a)
summary(Cox_8t)
#Isolation
Cox_1t = coxph(Surv(time_any, anyanyinf) ~ age+sex+factor(isolated), data=da4a)
summary(Cox_1t)
Cox_2t = coxph(Surv(time_any, anyanyinf) ~ age+factor(sex)+factor(white) +factor(isolated), data=da4a)
summary(Cox_2t)
Cox_3t = coxph(Surv(time_any, anyanyinf) ~ age+sex+white
+factor(edugroup)+deprivation_index+factor(isolated), data=da4a)
summary(Cox_3t)
Cox_4t = coxph(Surv(time_any, anyanyinf) ~ age+sex+white
+alcweekly+factor(bmi_who)+smoker+physical+factor(isolated), data=da4a)
summary(Cox_4t)
Cox_5t = coxph(Surv(time_any, anyanyinf) ~ age+sex+white +anyill+factor(isolated), data=da4a)
summary(Cox_5t)
Cox_6t = coxph(Surv(time_any, anyanyinf) ~ age+sex+white +dep_l2wb+factor(isolated), data=da4a)
summary(Cox_6t)
Cox_7t = coxph(Surv(time_any, anyanyinf) ~ age+sex+white +factor(crp_class)+factor(isolated), data=da4a)
summary(Cox_7t)
Cox_8t = coxph(Surv(time_any, anyanyinf) ~ age+sex+white
+factor(edugroup)+deprivation_index+alcweekly+factor(bmi_who)+smoker+physical+anyill+dep_l2w+factor(cr
p_class)+factor(isolated), data=da4a)
summary(Cox_8t)



Figures not used
#-------------------------------------- Loneliness----------------------------------------------------#
Cox_1a = coxph(Surv(time_any, anyanyinf) ~ age+sex+lonelyd, data=da4a)
Cox_1b = coxph(Surv(time_any, anyanyinf) ~ age+sex+white+lonelyd, data=da4a)
```

```
Cox_1c = coxph(Surv(time_any, anyanyinf) ~ age+sex+ white+edugroup+deprivation_index+lonelyd,
data=da4a)
Cox_1d = coxph(Surv(time_any, anyanyinf) ~ age+sex+ white+alcweekly+bmi+smoker+physical+lonelyd,
data=da4a)
Cox_1e = coxph(Surv(time_any, anyanyinf) ~ age+sex+ white+anyill+lonelyd, data=da4a)
Cox_1f = coxph(Surv(time_any, anyanyinf) ~ age+sex+ white+crp_over+lonelyd, data=da4a)
Cox_1g = coxph(Surv(time_any, anyanyinf) ~ age+sex+ white+dep_l2wb+lonelyd, data=da4a)
summary(Cox_1f)
Cox_1h = coxph(Surv(time_any, anyanyinf) ~ age+sex+white
+edugroup+deprivation_index+alcweekly+bmi+smoker+physical+anyill+dep_l2w+crp_over+lonelyd,
data=da4a)
summary(Cox_1h)
Cox_1i = coxph(Surv(short_time, short_anyinf) ~ age+sex+lonelyd, data=da4a)
summary(Cox_1i)
Cox_1j = coxph(Surv(long_time, anyanyinf) ~ age+sex+lonelyd, data=da5a)
summary(Cox_1j)
Cox_1k = coxph( Surv(time_any, anyanyinf) ~ age+sex+lonelyd, data=da4d)
summary(Cox_1k)


ti1<- tidy(Cox_1a, conf.int=TRUE,  exponentiate = TRUE)
ti1 <- ti1 %>%  dplyr::select(terma =term, estimatea = estimate, conf.lowa =conf.low, conf.higha = conf.high,
p.valuea = p.value) %>% slice_tail()
ti2<- tidy(Cox_1b, conf.int=TRUE,  exponentiate = TRUE)
ti2 <- ti2 %>%  dplyr::select(terma =term, estimatea = estimate, conf.lowa =conf.low, conf.higha = conf.high,
p.valuea = p.value) %>% slice_tail()
ti3<- tidy(Cox_1c, conf.int=TRUE,  exponentiate = TRUE)
ti3 <- ti3 %>%  dplyr::select(terma =term, estimatea = estimate, conf.lowa =conf.low, conf.higha = conf.high,
p.valuea = p.value) %>% slice_tail()
ti4<- tidy(Cox_1d, conf.int=TRUE,  exponentiate = TRUE)
ti4 <- ti4 %>%  dplyr::select(terma =term, estimatea = estimate, conf.lowa =conf.low, conf.higha = conf.high,
p.valuea = p.value) %>% slice_tail()
ti5<- tidy(Cox_1e, conf.int=TRUE,  exponentiate = TRUE)
ti5 <- ti5 %>%  dplyr::select(terma =term, estimatea = estimate, conf.lowa =conf.low, conf.higha = conf.high,
p.valuea = p.value) %>% slice_tail()
ti6<- tidy(Cox_1f, conf.int=TRUE,  exponentiate = TRUE)
ti6 <- ti6 %>%  dplyr::select(terma =term, estimatea = estimate, conf.lowa =conf.low, conf.higha = conf.high,
p.valuea = p.value) %>% slice_tail()
ti7<- tidy(Cox_1g, conf.int=TRUE,  exponentiate = TRUE)
ti7 <- ti7 %>%  dplyr::select(terma =term, estimatea = estimate, conf.lowa =conf.low, conf.higha = conf.high,
p.valuea = p.value) %>% slice_tail()
ti8<- tidy(Cox_1h, conf.int=TRUE,  exponentiate = TRUE)
ti8 <- ti8 %>%  dplyr::select(terma =term, estimatea = estimate, conf.lowa =conf.low, conf.higha = conf.high,
p.valuea = p.value) %>% slice_tail()
ti9<- tidy(Cox_1i, conf.int=TRUE,  exponentiate = TRUE)
ti9 <- ti9 %>%  dplyr::select(terma =term, estimatea = estimate, conf.lowa =conf.low, conf.higha = conf.high,
p.valuea = p.value) %>% slice_tail()
ti10<- tidy(Cox_1j, conf.int=TRUE,  exponentiate = TRUE)
ti10 <- ti10 %>%  dplyr::select(terma =term, estimatea = estimate, conf.lowa =conf.low, conf.higha = conf.high,
p.valuea = p.value) %>% slice_tail()
ti11<- tidy(Cox_1k, conf.int=TRUE,  exponentiate = TRUE)
ti11 <- ti11 %>%  dplyr::select(terma =term, estimatea = estimate, conf.lowa =conf.low, conf.higha = conf.high,
p.valuea = p.value) %>% slice_tail()

new.row0 <- data.frame(terma="Follow-up", estimatea= NA,conf.lowa =NA, conf.higha =NA, p.valuea=
NA,stringsAsFactors=F)
```

```
new.row1 <- data.frame(terma="Exclusion", estimatea= NA,conf.lowa =NA, conf.higha =NA, p.valuea= NA,
stringsAsFactors=F)

ti9 <- rbind(new.row0, ti9)
ti11 <- rbind(new.row1, ti11)
tii <- rbind(ti1, ti2, ti3, ti4, ti5, ti6, ti7, ti8, ti9, ti10, ti11)

tii[1,1]<- "None"
tii[2,1]<- "Ethinicity"
tii[3,1]<- "Socioeconomic factors"
tii[4,1]<- "Health behaviours"
tii[5,1]<- "Long term illness"
tii[6,1]<- "CRP over 3 mg/l"
tii[7,1]<- "Depressive mood"
tii[8,1]<- "All Above"
tii[9,1]<- ""
tii[10,1]<- "The first 3 years"
tii[11,1]<- "From year 3 onwards"
tii[12,1]<- ""
tii[13,1]<- "Any chronic disease \nhospitalisation"
tii[1,6]<- NA
tii[2,6] <- (tii[1,2]-tii[2,2])/(tii[1,2]-1)
tii[3,6] <- (tii[1,2]-tii[3,2])/(tii[1,2]-1)
tii[4,6] <- (tii[1,2]-tii[4,2])/(tii[1,2]-1)
tii[5,6] <- (tii[1,2]-tii[5,2])/(tii[1,2]-1)
tii[6,6] <- (tii[1,2]-tii[6,2])/(tii[1,2]-1)
tii[7,6] <- (tii[1,2]-tii[7,2])/(tii[1,2]-1)
tii[8,6] <- (tii[1,2]-tii[8,2])/(tii[1,2]-1)
tii[10,6] <- (tii[1,2]-tii[10,2])/(tii[1,2]-1)
tii[11,6] <- (tii[1,2]-tii[11,2])/(tii[1,2]-1)
tii[13,6] <- (tii[1,2]-tii[13,2])/(tii[1,2]-1)

colnames(tii)[6] <- c("Perm")

tii[1,7]<-  paste(as.character(glance(Cox_1a)[1]))
tii[2,7]<-  paste(as.character(glance(Cox_1b)[1]))
tii[3,7]<-  paste(as.character(glance(Cox_1c)[1]))
tii[4,7]<-  paste(as.character(glance(Cox_1d)[1]))
tii[5,7]<-  paste(as.character(glance(Cox_1e)[1]))
tii[6,7]<-  paste(as.character(glance(Cox_1f)[1]))
tii[7,7]<-  paste(as.character(glance(Cox_1g)[1]))
tii[8,7]<-  paste(as.character(glance(Cox_1h)[1]))
tii[10,7]<- paste(as.character(glance(Cox_1i)[1]))
tii[11,7]<- paste(as.character(glance(Cox_1j)[1]))
tii[13,7]<- paste(as.character(glance(Cox_1k)[1]))

tii[1,8]<-  paste(as.character(glance(Cox_1a)[2]))
tii[2,8]<-  paste(as.character(glance(Cox_1b)[2]))
tii[3,8]<-  paste(as.character(glance(Cox_1c)[2]))
tii[4,8]<-  paste(as.character(glance(Cox_1d)[2]))
tii[5,8]<-  paste(as.character(glance(Cox_1e)[2]))
tii[6,8]<-  paste(as.character(glance(Cox_1f)[2]))
tii[7,8]<-  paste(as.character(glance(Cox_1g)[2]))
tii[8,8]<-  paste(as.character(glance(Cox_1h)[2]))
tii[10,8]<- paste(as.character(glance(Cox_1i)[2]))
tii[11,8]<- paste(as.character(glance(Cox_1j)[2]))
tii[13,8]<- paste(as.character(glance(Cox_1k)[2]))
```

```
colnames(tii)[7] <- c("ncas")
colnames(tii)[8] <- c("ncases")
par(family = "Times New Roman")
rowseq <- seq(nrow(tii),1)
par(mai=c(1,0,0,0))
par(omi = c(0, 0, 0, 0)) # Outer margins back
plot(tii$estimatea, rowseq, pch=15,cex = 1.5,
    xlim=c(1,1), ylim=c(0,17),
    xlab='', ylab='', yaxt='n', xaxt='n',
    bty='n', log="x")
axis(1, round(log(seq(0.6,3,by=0.1))+1, 1), cex.axis=1.1, font=2)
segments(1,-1,1, 13, lty=3, lwd=1.5)
segments(tii$conf.lowa, rowseq, tii$conf.higha, rowseq, cex=4, lwd=2)

text(0.1,(nrow(tii)+3.7), "A.", cex=1.2, font=2, pos=4)

text(0.1,(nrow(tii)-8), "Follow-up", cex=1.2, font=2, pos=4)
text(0.1,(nrow(tii)+2.7), "Loneliness", cex=1.2, font=2, pos=4)
text(0.1,(nrow(tii)+1.7), "Study population", cex=1.2, font=2, pos=4)
text(0.1,(nrow(tii)+1), "Adjusted in addition to age and sex", cex=1.2, font=2, pos=4)
text(0.1,(nrow(tii)-11), "Exclusion", cex=1.2, font=2, pos=4)
t1h <- tii$terma
text(0.1,rowseq, t1h, cex=1.2, pos=4, font=1)

text(0.7,(nrow(tii)+1.7), "Hazard ratio (95% confidence interval) for hospital treated infection", cex=1.2,
font=2, pos=4)

t3b <- with(tii, paste(sprintf("%.2f",estimatea, 2),' (',sprintf("%.2f",conf.lowa, 2),' to ',sprintf("%.2f",conf.higha,
2),')',sep=''))
t3 <- gsub("(NA to NA)","", t3b)
t3 <- gsub("NA ()", "", t3)
t3 <- gsub("\\()", "", t3)
text(1.9,rowseq, t3, cex=1.2, font =1, pos=4)

text(0.30,(nrow(tii)+1.7), "N(total)", cex=1.2, font=2, pos=4)
#t5 <- with(tii, paste(round(Perm, 2)))
t6 <- with(tii, paste(ncas))
t6 <- gsub("NA", "", t6)
text(0.30,rowseq, t6, cex=1.2, font =1, pos=4)

text(0.40,(nrow(tii)+1.7), "N(cases)", cex=1.2, font=2, pos=4)
t7 <- with(tii, paste(ncases))
t7 <- gsub("NA", "", t7)
text(0.40,rowseq, t7, cex=1.2, font =1, pos=4)

segments(0.1, 13.5, 5.5,13.5   , lty=1, lwd=1.2)

dev.print(tiff , "/Users/melovain/Documents/kone/Akatemia20/UKBB_Infl/LPH_rew/Kuvat/Figure2a_b", res =
100, height = 10, width = 19, units
       = "in", compression = "zip")

#-------------------------------------- Isolation ----------------------------------------------------#
Cox_1a = coxph(Surv(time_any, anyanyinf) ~ age+sex+isolated, data=da4a)
Cox_1b = coxph(Surv(time_any, anyanyinf) ~ age+sex+white+isolated, data=da4a)
```

```
Cox_1c = coxph(Surv(time_any, anyanyinf) ~ age+sex+white+edugroup+deprivation_index+isolated,
data=da4a)
Cox_1d = coxph(Surv(time_any, anyanyinf) ~ age+sex+ white+alcweekly+bmi+smoker+physical+isolated,
data=da4a)
Cox_1e = coxph(Surv(time_any, anyanyinf) ~ age+sex+ white+anyill+isolated, data=da4a)
Cox_1f = coxph(Surv(time_any, anyanyinf) ~ age+sex+ white+crp_over+isolated, data=da4a)
Cox_1g = coxph(Surv(time_any, anyanyinf) ~ age+sex+ white+dep_l2wb+isolated, data=da4a)
summary(Cox_1f)
Cox_1h = coxph(Surv(time_any, anyanyinf) ~ age+sex+white
+edugroup+deprivation_index+alcweekly+bmi+smoker+physical+anyill+dep_l2w+crp_over+isolated,
data=da4a)
summary(Cox_1h)
Cox_1i = coxph(Surv(short_time, short_anyinf) ~ age+sex+isolated, data=da4a)
summary(Cox_1i)
Cox_1j = coxph(Surv(long_time, anyanyinf) ~ age+sex+isolated, data=da5a)
summary(Cox_1j)
Cox_1k = coxph( Surv(time_any, anyanyinf) ~ age+sex+isolated, data=da4d)
summary(Cox_1k)

ti1<- tidy(Cox_1a, conf.int=TRUE,  exponentiate = TRUE)
ti1 <- ti1 %>%  dplyr::select(terma =term, estimatea = estimate, conf.lowa =conf.low, conf.higha = conf.high,
p.valuea = p.value) %>% slice_tail()
ti2<- tidy(Cox_1b, conf.int=TRUE,  exponentiate = TRUE)
ti2 <- ti2 %>%  dplyr::select(terma =term, estimatea = estimate, conf.lowa =conf.low, conf.higha = conf.high,
p.valuea = p.value) %>% slice_tail()
ti3<- tidy(Cox_1c, conf.int=TRUE,  exponentiate = TRUE)
ti3 <- ti3 %>%  dplyr::select(terma =term, estimatea = estimate, conf.lowa =conf.low, conf.higha = conf.high,
p.valuea = p.value) %>% slice_tail()
ti4<- tidy(Cox_1d, conf.int=TRUE,  exponentiate = TRUE)
ti4 <- ti4 %>%  dplyr::select(terma =term, estimatea = estimate, conf.lowa =conf.low, conf.higha = conf.high,
p.valuea = p.value) %>% slice_tail()
ti5<- tidy(Cox_1e, conf.int=TRUE,  exponentiate = TRUE)
ti5 <- ti5 %>%  dplyr::select(terma =term, estimatea = estimate, conf.lowa =conf.low, conf.higha = conf.high,
p.valuea = p.value) %>% slice_tail()
ti6<- tidy(Cox_1f, conf.int=TRUE,  exponentiate = TRUE)
ti6 <- ti6 %>%  dplyr::select(terma =term, estimatea = estimate, conf.lowa =conf.low, conf.higha = conf.high,
p.valuea = p.value) %>% slice_tail()
ti7<- tidy(Cox_1g, conf.int=TRUE,  exponentiate = TRUE)
ti7 <- ti7 %>%  dplyr::select(terma =term, estimatea = estimate, conf.lowa =conf.low, conf.higha = conf.high,
p.valuea = p.value) %>% slice_tail()
ti8<- tidy(Cox_1h, conf.int=TRUE,  exponentiate = TRUE)
ti8 <- ti8 %>%  dplyr::select(terma =term, estimatea = estimate, conf.lowa =conf.low, conf.higha = conf.high,
p.valuea = p.value) %>% slice_tail()
ti9<- tidy(Cox_1i, conf.int=TRUE,  exponentiate = TRUE)
ti9 <- ti9 %>%  dplyr::select(terma =term, estimatea = estimate, conf.lowa =conf.low, conf.higha = conf.high,
p.valuea = p.value) %>% slice_tail()
ti10<- tidy(Cox_1j, conf.int=TRUE,  exponentiate = TRUE)
ti10 <- ti10 %>%  dplyr::select(terma =term, estimatea = estimate, conf.lowa =conf.low, conf.higha = conf.high,
p.valuea = p.value) %>% slice_tail()
ti11<- tidy(Cox_1k, conf.int=TRUE,  exponentiate = TRUE)
ti11 <- ti11 %>%  dplyr::select(terma =term, estimatea = estimate, conf.lowa =conf.low, conf.higha = conf.high,
p.valuea = p.value) %>% slice_tail()

new.row0 <- data.frame(terma="Follow-up", estimatea= NA,conf.lowa =NA, conf.higha =NA, p.valuea=
NA,stringsAsFactors=F)
new.row1 <- data.frame(terma="Exclusion", estimatea= NA,conf.lowa =NA, conf.higha =NA, p.valuea= NA,
stringsAsFactors=F)
```

```
ti9 <- rbind(new.row0, ti9)
ti11 <- rbind(new.row1, ti11)
tii <- rbind(ti1, ti2, ti3, ti4, ti5, ti6, ti7, ti8, ti9, ti10, ti11)

tii[1,1]<- "None"
tii[2,1]<- "Ethinicity"
tii[3,1]<- "Socioeconomic factors"
tii[4,1]<- "Health behaviours"
tii[5,1]<- "Long term illness"
tii[6,1]<- "CRP over 3 mg/l"
tii[7,1]<- "Depressive mood"
tii[8,1]<- "All Above"
tii[9,1]<- ""
tii[10,1]<- "The first 3 years"
tii[11,1]<- "From year 3 onwards"
tii[12,1]<- ""
tii[13,1]<- "Any chronic disease \nhospitalisation"
tii[1,6]<- NA
tii[2,6] <- (tii[1,2]-tii[2,2])/(tii[1,2]-1)
tii[3,6] <- (tii[1,2]-tii[3,2])/(tii[1,2]-1)
tii[4,6] <- (tii[1,2]-tii[4,2])/(tii[1,2]-1)
tii[5,6] <- (tii[1,2]-tii[5,2])/(tii[1,2]-1)
tii[6,6] <- (tii[1,2]-tii[6,2])/(tii[1,2]-1)
tii[7,6] <- (tii[1,2]-tii[7,2])/(tii[1,2]-1)
tii[8,6] <- (tii[1,2]-tii[8,2])/(tii[1,2]-1)
tii[10,6] <- (tii[1,2]-tii[10,2])/(tii[1,2]-1)
tii[11,6] <- (tii[1,2]-tii[11,2])/(tii[1,2]-1)
tii[13,6] <- (tii[1,2]-tii[13,2])/(tii[1,2]-1)

colnames(tii)[6] <- c("Perm")

tii[1,7]<-  paste(as.character(glance(Cox_1a)[1]))
tii[2,7]<-  paste(as.character(glance(Cox_1b)[1]))
tii[3,7]<-  paste(as.character(glance(Cox_1c)[1]))
tii[4,7]<-  paste(as.character(glance(Cox_1d)[1]))
tii[5,7]<-  paste(as.character(glance(Cox_1e)[1]))
tii[6,7]<-  paste(as.character(glance(Cox_1f)[1]))
tii[7,7]<-  paste(as.character(glance(Cox_1g)[1]))
tii[8,7]<-  paste(as.character(glance(Cox_1h)[1]))
tii[10,7]<- paste(as.character(glance(Cox_1i)[1]))
tii[11,7]<- paste(as.character(glance(Cox_1j)[1]))
tii[13,7]<- paste(as.character(glance(Cox_1k)[1]))

tii[1,8]<-  paste(as.character(glance(Cox_1a)[2]))
tii[2,8]<-  paste(as.character(glance(Cox_1b)[2]))
tii[3,8]<-  paste(as.character(glance(Cox_1c)[2]))
tii[4,8]<-  paste(as.character(glance(Cox_1d)[2]))
tii[5,8]<-  paste(as.character(glance(Cox_1e)[2]))
tii[6,8]<-  paste(as.character(glance(Cox_1f)[2]))
tii[7,8]<-  paste(as.character(glance(Cox_1g)[2]))
tii[8,8]<-  paste(as.character(glance(Cox_1h)[2]))
tii[10,8]<- paste(as.character(glance(Cox_1i)[2]))
tii[11,8]<- paste(as.character(glance(Cox_1j)[2]))
tii[13,8]<- paste(as.character(glance(Cox_1k)[2]))
```

```
colnames(tii)[7] <- c("ncas")
colnames(tii)[8] <- c("ncases")
par(family = "Times New Roman")
rowseq <- seq(nrow(tii),1)
par(mai=c(1,0,0,0))
par(omi = c(0, 0, 0, 0)) # Outer margins back

plot(tii$estimatea, rowseq, pch=15,cex = 1.5,
    xlim=c(1,1), ylim=c(0,17),
    xlab='', ylab='', yaxt='n', xaxt='n',
    bty='n', log="x")
axis(1, round(log(seq(0.6,3,by=0.1))+1, 1), cex.axis=1.1, font=2)
segments(1,-1,1, 13, lty=3, lwd=1.5)
segments(tii$conf.lowa, rowseq, tii$conf.higha, rowseq, cex=4, lwd=2)

text(0.1,(nrow(tii)+3.7), "B.", cex=1.2, font=2, pos=4)

text(0.1,(nrow(tii)-8), "Follow-up", cex=1.2, font=2, pos=4)
text(0.1,(nrow(tii)+2.7), "Isolated", cex=1.2, font=2, pos=4)
text(0.1,(nrow(tii)+1.7), "Study population", cex=1.2, font=2, pos=4)
text(0.1,(nrow(tii)+1), "Adjusted in addition to age and sex", cex=1.2, font=2, pos=4)
text(0.1,(nrow(tii)-11), "Exclusion", cex=1.2, font=2, pos=4)
t1h <- tii$terma
text(0.1,rowseq, t1h, cex=1.2, pos=4, font=1)

text(0.7,(nrow(tii)+1.7), "Hazard ratio (95% confidence interval) for hospital treated infection", cex=1.2,
font=2, pos=4)

t3b <- with(tii, paste(sprintf("%.2f",estimatea, 2),' (',sprintf("%.2f",conf.lowa, 2),' to ',sprintf("%.2f",conf.higha,
2),')',sep=''))
t3 <- gsub("(NA to NA)","", t3b)
t3 <- gsub("NA ()", "", t3)
t3 <- gsub("\\()", "", t3)
text(1.9,rowseq, t3, cex=1.2, font =1, pos=4)

text(0.30,(nrow(tii)+1.7), "N(total)", cex=1.2, font=2, pos=4)
t6 <- with(tii, paste(ncas))
t6 <- gsub("NA", "", t6)
text(0.30,rowseq, t6, cex=1.2, font =1, pos=4)

text(0.40,(nrow(tii)+1.7), "N(cases)", cex=1.2, font=2, pos=4)
t7 <- with(tii, paste(ncases))
t7 <- gsub("NA", "", t7)
text(0.40,rowseq, t7, cex=1.2, font =1, pos=4)

segments(0.1, 13.5, 5.5,13.5   , lty=1, lwd=1.2)

dev.print(tiff , "/Users/melovain/Documents/kone/Akatemia20/UKBB_Infl/LPH_rew/Kuvat/Figure2b_b", res =
100, height = 10, width = 19, units
        = "in", compression = "zip")

# --------------- Flowchart in flowchart-file
# --------------- Hessup results n Hessup -file
# --------------- Excluded other diseases in Other_disease -file
#---------------  All other ICD-10-categories ---------------#

load("/Users/melovain/Documents/kone/Akatemia20/UKBB_Infl/data_o.Rdata")
```

```
dlog4 <- left_join(da4a, data_o, by =c("id"))
names(data_o)

dlog4$cancer <- replace0(dlog4$cancer)
dlog4$blood <- replace0(dlog4$blood)
dlog4$endocrine <- replace0(dlog4$endocrine)
dlog4$mental <- replace0(dlog4$mental)
dlog4$nervous <- replace0(dlog4$nervous)
dlog4$eye_ear <- replace0(dlog4$eye_ear)
dlog4$circulatory <- replace0(dlog4$circulatory)
dlog4$respiratory <- replace0(dlog4$respiratory)
dlog4$digestive <- replace0(dlog4$digestive)
dlog4$skin <- replace0(dlog4$skin)
dlog4$musculo <- replace0(dlog4$musculo)
dlog4$genoto <- replace0(dlog4$genoto)

dlog4l <- dlog4 %>% drop_na(white, lonelyd)
dlog4i <- dlog4 %>% drop_na(white, isolated)


summary(glm(cancer~sex + age + white + lonelyd,data= dlog4,family="binomial"))
summary(glm(anyanyinf~sex + age + white + lonelyd,data= dlog4,family="binomial"))

koe4l<-dlog4 %>%
drop_na(sex,age,white,edugroup,deprivation_index,alcweekly,bmi,smoker,physical,anyill,dep_l2w,crp_over,lo
nelyd)
koe4i<-dlog4 %>%
drop_na(sex,age,white,edugroup,deprivation_index,alcweekly,bmi,smoker,physical,anyill,dep_l2w,crp_over,is
olated)

library(broom)
library(purrr)
# variables <- n<-
as.character(expression(cancer,blood,endocrine,mental,nervous,eye_ear,circulator,respirator,digestive,skin,m
usculo,genoto))
# cat(paste0('"', paste(n, collapse="~\", \""), '~"'))

dep <- list("cancer~", "blood~", "endocrine~", "mental~", "nervous~", "eye_ear~", "circulatory~",
"respiratory~", "digestive~", "skin~", "musculo~", "genoto~")
# dep<-list("mpg~","cyl~","disp~") # list of unique dependent variables with ~
# indep1<-list("hp","drat","wt")  # list of first unique independent variables
# indep2<-list("qsec","vs","am") # list of second unique independent variables
# Map(function(x,y,z) lm(as.formula(paste(x,paste(list(y,z),collapse="+"))),data=mtcars),dep,indep1,indep2)

sum(is.na(dlog4$sex))
#------------------------------------------ Loneliness ---------------------------------------------#
models2 <-dep %>%  str_c(.,"sex + age + white +
edugroup+deprivation_index+alcweekly+bmi+smoker+physical+anyill+dep_l2w+crp_over+lonelyd") %>%
  map(
   .f = ~glm(
     formula = as.formula(.x),
     data= dlog4,
     family="binomial")) %>%
  map(
   .f = ~tidy(
    .x,
```

```
    exponentiate = TRUE,
    conf.int = TRUE)) %>%
  bind_rows() %>%
  mutate(across(where(is.numeric), round, digits = 4))

glance(glm(cancer~sex + age + white +
edugroup+deprivation_index+alcweekly+bmi+smoker+physical+anyill+dep_l2wb+crp_over+lonelyd,data=
dlog4,family="binomial"))

# modelsn <-dep %>%  str_c(.,"sex + age + white +
edugroup+deprivation_index+alcweekly+bmi+smoker+physical+anyill+dep_l2w+crp_over+lonelyd") %>%
#   map(
#     .f = ~glm(
#       formula = as.formula(.x),
#       data= dlog4,
#       family="binomial")) %>%
#   map(
#     .f = ~glance(
#       .x)) %>%
#   bind_rows() %>% select(nobs) %>%
#   mutate(across(where(is.numeric), round, digits = 2))
 models2 <- models2 %>% filter(term %in% c("lonelyd"))
# models2m <- cbind(models2m, modelsn)
models2
mm<-dplyr::add_row(models2,term="Disease outcome", .before = 1)

mm[2,1] <- "Cancer"


mm[3,1] <- "Diseases_of_the_blood"
mm[4,1] <- "Endocrine_diseases"
mm[5,1] <- "Mental_and_behavioral"
mm[6,1] <- "Diseases_of_the_nervous_system"
mm[7,1] <- "Diseases_of_the_eye_ear"
mm[8,1] <- "Diseases_of_the_circulatory_systems"
mm[9,1] <- "Diseases_of_the_respiratory_systems"
mm[10,1] <- "Diseases_of_the_digestive_systems"
mm[11,1] <- "Diseases_of_the_skin"
mm[12,1] <- "Diseases_of_the_musculoskeletal_system"
mm[13,1] <- "Diseases_of_the_genitourinary_system"
mm<-mm %>% select("N(total=351 439)"= term, OddsRatio =estimate, LowerCL=conf.low, HigherCL=conf.high,
"P-value"=p.value)
mm<-tibble::add_column(mm,"N(cases)"=NA, .before = 2)
mm
mm[2,2] <- frq(koe4l$cancer)[[1]]$frq[2]
mm[3,2] <- frq(koe4l$blood)[[1]]$frq[2]
mm[4,2] <- frq(koe4l$endocrine)[[1]]$frq[2]
mm[5,2] <- frq(koe4l$mental)[[1]]$frq[2]
mm[6,2] <- frq(koe4l$nervous)[[1]]$frq[2]
mm[7,2] <- frq(koe4l$eye_ear)[[1]]$frq[2]
mm[8,2] <- frq(koe4l$circulatory)[[1]]$frq[2]
mm[9,2] <- frq(koe4l$respiratory)[[1]]$frq[2]
mm[10,2] <-frq(koe4l$digestive)[[1]]$frq[2]
mm[11,2] <-frq(koe4l$skin)[[1]]$frq[2]
mm[12,2] <-frq(koe4l$musculo)[[1]]$frq[2]
mm[13,2] <-frq(koe4l$genoto)[[1]]$frq[2]
mm <-mm %>%  unite("95%CI", LowerCL, HigherCL, sep = "-")
```

```
tab_df(mm, title="Table 2. Association (multivariable adjusted) between loneliness and risk of hospital-treated
disease")

#------------------------------------------ Isolation ---------------------------------------------#
models2 <-dep %>%  str_c(.,"sex + age + white +
edugroup+deprivation_index+alcweekly+bmi+smoker+physical+anyill+dep_l2w+crp_over+isolated") %>%
  map(
    .f = ~glm(
      formula = as.formula(.x),
      data= dlog4,
      family="binomial")) %>%
  map(
    .f = ~tidy(
      .x,
      exponentiate = TRUE,
      conf.int = TRUE)) %>%
  bind_rows() %>%
  mutate(across(where(is.numeric), round, digits = 4))

glance(glm(cancer~sex + age + white +
edugroup+deprivation_index+alcweekly+bmi+smoker+physical+anyill+dep_l2w+crp_over+isolated,data=
dlog4,family="binomial"))

# modelsn <-dep %>%  str_c(.,"sex + age + white +
edugroup+deprivation_index+alcweekly+bmi+smoker+physical+anyill+dep_l2w+crp_over+isolated") %>%
#   map(
#     .f = ~glm(
#       formula = as.formula(.x),
#       data= dlog4,
#       family="binomial")) %>%
#   map(
#     .f = ~glance(
#       .x)) %>%
#   bind_rows() %>% select(nobs) %>%
#   mutate(across(where(is.numeric), round, digits = 2))
 models2 <- models2 %>% filter(term %in% c("isolated"))
# models2m <- cbind(models2m, modelsn)
# models2m
mm<-dplyr::add_row(models2,term="Disease outcome", .before = 1)

mm[2,1] <- "Cancer"


mm[3,1] <- "Diseases_of_the_blood"
mm[4,1] <- "Endocrine_diseases"
mm[5,1] <- "Mental_and_behavioral"
mm[6,1] <- "Diseases_of_the_nervous_system"
mm[7,1] <- "Diseases_of_the_eye_ear"
mm[8,1] <- "Diseases_of_the_circulatory_systems"
mm[9,1] <- "Diseases_of_the_respiratory_systems"
mm[10,1] <- "Diseases_of_the_digestive_systems"
mm[11,1] <- "Diseases_of_the_skin"
mm[12,1] <- "Diseases_of_the_musculoskeletal_system"
mm[13,1] <- "Diseases_of_the_genitourinary_system"
mm<-mm %>% select("N(total=360 809)"= term, OddsRatio =estimate, LowerCL=conf.low, HigherCL=conf.high,
"P-value"=p.value)
mm<-tibble::add_column(mm,"N(cases)"=NA, .before = 2)
```

```
mm
mm[2,2] <- frq(koe4i$cancer)[[1]]$frq[2]
mm[3,2] <- frq(koe4i$blood)[[1]]$frq[2]
mm[4,2] <- frq(koe4i$endocrine)[[1]]$frq[2]
mm[5,2] <- frq(koe4i$mental)[[1]]$frq[2]
mm[6,2] <- frq(koe4i$nervous)[[1]]$frq[2]
mm[7,2] <- frq(koe4i$eye_ear)[[1]]$frq[2]
mm[8,2] <- frq(koe4i$circulatory)[[1]]$frq[2]
mm[9,2] <- frq(koe4i$respiratory)[[1]]$frq[2]
mm[10,2] <-frq(koe4i$digestive)[[1]]$frq[2]
mm[11,2] <-frq(koe4i$skin)[[1]]$frq[2]
mm[12,2] <-frq(koe4i$musculo)[[1]]$frq[2]
mm[13,2] <-frq(koe4i$genoto)[[1]]$frq[2]
mm <-mm %>%  unite("95%CI", LowerCL, HigherCL, sep = "-")
tab_df(mm, title="Table 2. Association (multivariable adjusted) between isolation and risk of hospital-treated
disease")
#---------------------------------------- get the specific inflammations ----------------------------------------#
load("icd_data1.Rdata")
icd1<- icd_data1 %>%  pivot_longer(!id, names_to = "ICD", values_to = "diagnose")%>% drop_na(diagnose)
icd1 <- icd1 %>% arrange(id, ICD)

koe <- icd1 %>% filter(diagnose!="")
diag_1<- koe %>% dplyr::filter(substr(diagnose,1,3) == "A00" | substr(diagnose,1,3) == "A01" |
substr(diagnose,1,3) == "A02" |
                        substr(diagnose,1,3) == "A03" | substr(diagnose,1,3) == "A04" | substr(diagnose,1,3) ==
"A05" |
                        substr(diagnose,1,3) == "A06" | substr(diagnose,1,3) == "A07" | substr(diagnose,1,3) ==
"A08" | substr(diagnose,1,3) == "A09") %>%
                        group_by(id) %>% arrange(id, ICD) %>% filter(row_number()==1) %>% mutate(gastro=1)
diag_2 <- koe %>% dplyr::filter(substr(diagnose,1,3) =="J09" | substr(diagnose,1,3) =="J10" |
                    substr(diagnose,1,3) =="J11") %>% group_by(id) %>% arrange(id, ICD) %>%
filter(row_number()==1) %>% mutate(influ=1)
diag_3 <- koe %>% dplyr::filter(substr(diagnose,1,3) =="J12" | substr(diagnose,1,3) =="J13" |
                             substr(diagnose,1,3) =="J14" | substr(diagnose,1,3) =="J15" |
                             substr(diagnose,1,3) =="J16" | substr(diagnose,1,3) =="J17" |
                             substr(diagnose,1,3) =="J18") %>%
                         group_by(id) %>% arrange(id, ICD) %>% filter(row_number()==1) %>% mutate(pneumo=1)
diag_4 <- koe %>% dplyr::filter(substr(diagnose,1,3) =="N10" | substr(diagnose,1,5) =="N136" |
                              substr(diagnose,1,5) =="N151" | substr(diagnose,1,5) =="N300" |
                              substr(diagnose,1,5) =="N390") %>% group_by(id) %>% arrange(id, ICD) %>%
filter(row_number()==1) %>% mutate(urinary=1)
diag_5 <- koe %>% dplyr::filter(substr(diagnose,1,3) =="A46"  | substr(diagnose,1,3) =="L00" |
                         substr(diagnose,1,3) =="L01" | substr(diagnose,1,3) =="L02" |
                         substr(diagnose,1,3) =="L03" | substr(diagnose,1,3) =="L04" |
                         substr(diagnose,1,3) =="L05" | substr(diagnose,1,3) =="L06" |
                         substr(diagnose,1,3) =="L07" | substr(diagnose,1,3) =="L08") %>%
                    group_by(id) %>% arrange(id, ICD) %>% filter(row_number()==1) %>% mutate(skin=1)
diag_6 <- koe %>% dplyr::filter(substr(diagnose,1,3) == "J09"  | substr(diagnose,1,3) =="J10" |
                        substr(diagnose,1,3) =="J11" | substr(diagnose,1,3) =="J12" |
                        substr(diagnose,1,3) =="J171" ) %>%
                         group_by(id) %>% arrange(id, ICD) %>% filter(row_number()==1) %>% mutate(influ_vp=1)
diag_7 <- koe %>% dplyr::filter(substr(diagnose,1,3) == "J13"  | substr(diagnose,1,3) =="J14" |
                         substr(diagnose,1,3) =="J15" | substr(diagnose,1,3) =="J160" |
                         substr(diagnose,1,3) =="J168" | substr(diagnose,1,3) =="J170" |
                        substr(diagnose,1,3) =="J18" ) %>%
                         group_by(id) %>% arrange(id, ICD) %>% filter(row_number()==1) %>%
mutate(b_pneumo=1)
```

```
diag_1[diag_1 %>% duplicated(),]
diag_2[diag_2 %>% duplicated(),]
diag_3[diag_3 %>% duplicated(),]
diag_4[diag_4 %>% duplicated(),]
diag_5[diag_5 %>% duplicated(),]
diag_6[diag_6 %>% duplicated(),]
diag_7[diag_7 %>% duplicated(),]

das1<- diag_1 %>% ungroup %>% select(id, Icda=ICD, diagnose_a=diagnose, gastro)
das2<- diag_2 %>% ungroup %>% select(id, Icda=ICD, diagnose_b=diagnose, influ)
das3<- diag_3 %>% ungroup %>% select(id, Icda=ICD, diagnose_c=diagnose, pneumo)
das4<- diag_4 %>% ungroup %>% select(id, Icda=ICD, diagnose_d=diagnose, urinary)
das5<- diag_5 %>% ungroup %>% select(id, Icda=ICD, diagnose_e=diagnose, skin)
das6<- diag_6 %>% ungroup %>% select(id, Icda=ICD, diagnose_e=diagnose, influ_vp)
das7<- diag_7 %>% ungroup %>% select(id, Icda=ICD, diagnose_e=diagnose, b_pneumo)

data_sp <- plyr::join_all(list(das1, das2,das3,das4,das5, das6, das7), by=c("id"), type='full')
#save(data_sp, file = "/Users/melovain/Documents/kone/Akatemia20/UKBB_Infl/data_sp.Rdata")
load("/Users/melovain/Documents/kone/Akatemia20/UKBB_Infl/data_sp.Rdata")

dlog5 <- left_join(da4a, data_sp, by =c("id"))
dlog5$gastro <- replace0(dlog5$gastro)
dlog5$influ <- replace0(dlog5$influ)
dlog5$pneumo <- replace0(dlog5$pneumo)
dlog5$urinary <- replace0(dlog5$urinary)
dlog5$skin <- replace0(dlog5$skin)
dlog5$influ_vp <- replace0(dlog5$influ_vp)
dlog5$b_pneumo <- replace0(dlog5$b_pneumo)

koe5l<-dlog5 %>%
drop_na(sex,age,white,edugroup,deprivation_index,alcweekly,bmi,smoker,physical,anyill,dep_l2w,crp_over,lo
nelyd)
koe5i<-dlog5 %>%
drop_na(sex,age,white,edugroup,deprivation_index,alcweekly,bmi,smoker,physical,anyill,dep_l2w,crp_over,is
olated)

library(broom)
library(purrr)

dep2 <- list("gastro~", "influ~", "pneumo~", "urinary~", "skin~", "influ_vp~", "b_pneumo~")

models2 <-dep2 %>%  str_c(.,"sex + age + white +
edugroup+deprivation_index+alcweekly+bmi+smoker+physical+anyill+dep_l2w+crp_over+lonelyd") %>%
  map(
    .f = ~glm(
      formula = as.formula(.x),
      data= dlog5,
      family="binomial")) %>%
  map(
    .f = ~tidy(
      .x,
      exponentiate = TRUE,
      conf.int = TRUE)) %>%
  bind_rows() %>%
  mutate(across(where(is.numeric), round, digits = 2))
```

```
models2 <- models2 %>% filter(term %in% c("lonelyd"))
# models2m <- cbind(models2m, modelsn)
# models2m
mm<-dplyr::add_row(models2,term="Disease outcome", .before = 1)

mm[2,1] <- "Gastrointestinal infections"


mm[3,1] <- "Influenza"
mm[4,1] <- "Pneumonia"
mm[5,1] <- "Urinary tract infections"
mm[6,1] <- "Skin and subcutaneous tissue infections"
mm[7,1] <- "Influenze and virus pneumonia"
mm[8,1] <- "Bacterial_pneumonia"

mm<-mm %>% select("N(total=351 439)"= term, OddsRatio =estimate, LowerCL=conf.low, HigherCL=conf.high,
"P-value"=p.value)
mm<-tibble::add_column(mm,"N(cases)"=NA, .before = 2)
mm
mm[2,2] <- frq(koe5l$gastro)[[1]]$frq[2]
mm[3,2] <- frq(koe5l$influ)[[1]]$frq[2]
mm[4,2] <- frq(koe5l$pneumo)[[1]]$frq[2]
mm[5,2] <- frq(koe5l$urinary)[[1]]$frq[2]
mm[6,2] <- frq(koe5l$skin)[[1]]$frq[2]
mm[7,2] <- frq(koe5l$influ_vp)[[1]]$frq[2]
mm[8,2] <- frq(koe5l$b_pneumo)[[1]]$frq[2]

mm <-mm %>%  unite("95%CI", LowerCL, HigherCL, sep = "-")
tab_df(mm, title="Table 2. Association (multivariable adjusted) between loneliness and risk of specific
hospital-treated disease")

models2 <-dep2 %>%  str_c(.,"sex + age + white +
edugroup+deprivation_index+alcweekly+bmi+smoker+physical+anyill+dep_l2w+crp_over+isolated") %>%
  map(
    .f = ~glm(
      formula = as.formula(.x),
      data= dlog5,
      family="binomial")) %>%
  map(
    .f = ~tidy(
      .x,
      exponentiate = TRUE,
      conf.int = TRUE)) %>%
  bind_rows() %>%
  mutate(across(where(is.numeric), round, digits = 2))

models2 <- models2 %>% filter(term %in% c("isolated"))
# models2m <- cbind(models2m, modelsn)
# models2m
mm<-dplyr::add_row(models2,term="Disease outcome", .before = 1)

mm[2,1] <- "Gastrointestinal infections"


mm[3,1] <- "Influenza"
mm[4,1] <- "Pneumonia"
mm[5,1] <- "Urinary tract infections"
```

```
mm[6,1] <- "Skin and subcutaneous tissue infections"
mm[7,1] <- "Influenze and virus pneumonia"
mm[8,1] <- "Bacterial_pneumonia"
mm<-mm %>% select("N(total=360 809)"= term, OddsRatio =estimate, LowerCL=conf.low, HigherCL=conf.high,
"P-value"=p.value)
mm<-tibble::add_column(mm,"N(cases)"=NA, .before = 2)
mm
mm[2,2] <- frq(koe5i$gastro)[[1]]$frq[2]
mm[3,2] <- frq(koe5i$influ)[[1]]$frq[2]
mm[4,2] <- frq(koe5i$pneumo)[[1]]$frq[2]
mm[5,2] <- frq(koe5i$urinary)[[1]]$frq[2]
mm[6,2] <- frq(koe5i$skin)[[1]]$frq[2]
mm[7,2] <- frq(koe5i$influ_vp)[[1]]$frq[2]
mm[8,2] <- frq(koe5i$b_pneumo)[[1]]$frq[2]

mm <-mm %>%  unite("95%CI", LowerCL, HigherCL, sep = "-")
tab_df(mm, title="Table 2. Association (multivariable adjusted) between isolation and risk of specific hospital-
treated disease")


library(paletteer)
library(gt)
library(scales)
mm %>%
  gt(rowname_col = "size") %>%
  tab_options(table.font.names = "Times") %>%
  data_color(columns = 3:5,
           colors = col_numeric(palette = c("white","red"),
                          domain = c(0.90,1.24))) %>%
  data_color(columns = 6,
           colors = col_numeric(palette = c("red","white"),
                          domain = c(0.00,0.72))) %>%
  tab_header(
    title = md("Table 2. Association (multivariable adjusted) between isolation and risk of hospital-treated
disease"))



dlog4 <- dlog4 %>% mutate(disease_tot= if_else(cancer==1 | blood endocrine mental nervous eye_ear
circulatory respiratory digestive skin musculogenoto)
))
  filter(!is.na(bill_length_mm)) %>%
  mutate(
    category = case_when(
      if_all(contains("bill"), big) ~ "both big",
      if_any(contains("bill"), big) ~ "one big",
      TRUE                        ~ "small"
    ))

 #-------------------------------------------------------------------#
 #                                                  #
 #----------------------Hessup- analyses ----------------------------#
 #                                                  #
 #-------------------------------------------------------------------#
 library(tidyverse)
 library(readstata13)
 library(sjmisc)
```

```
library(sjstats)
library(ggeffects)
library(haven)
library(broom)
library(sas7bdat)
library(janitor)
library(foreign)
library(readxl)
library(lubridate)
library(sjPlot)
ds2 <- dsos1 %>% select(id2, sex= SUKUPUOLI, edugroup_1= L2, dep_l2w_1 =beck_03, physical_1=
metday_03,smoker_1=smoking_03,alcweekly_1 = kaytto_03,anyill_1=Sairaudet_2003_lkm,
            isolated_1=tukiluok_03, isolated_2=tukiluok_12, birth_year= SVUOSI,bmi_1 = bmi_03, bmi_2 =
BMI_12,
            lonely_1=L101, lonely_2= M105, death_date=kuolinpvm)
ds2$year <- paste("19", ds2$birth_year)
ds2$year <- gsub(" ", "", ds2$year)
ds2$birth_date <- paste(ds2$year, "07-01",sep="-")
ds2$birth_date <- lubridate::date(ds2$birth_date)
ds2$death_date <- lubridate::date(ds2$death_date)
ds2 <- ds2 %>% mutate(dead= if_else(is.na(death_date), 0,1) )
 ds2 <- ds2 %>% mutate(death_date= if_else(dead==0,as.Date(ymd("2012-12-31")),death_date) )
ds2$time_dead <- as.numeric(difftime(ds2$death_date, as.Date(ymd("2002-12-31")), units = "weeks")/52)
ds2$age <- 2003-as.numeric(ds2$year)
library(epikit)
ds2 <- ds2 %>%
 mutate(
  age_group = age_categories(
   age,
   breakers = c(0, 31, 51, 61),
   ceiling = TRUE)) # 70 is ceiling, all above become NA
# show table
 ds2 <- ds2 %>%  rowwise %>%
 mutate(isolated = case_when(
  isolated_1==  1 ~ 1,
  isolated_1 == 2 ~ 0,
  isolated_1 == 3 ~ 0,
  isolated_1 == 4 ~ 0)) %>%
 mutate(lonelyd = case_when(
  lonely_1== 1 ~ 1,
  lonely_1== 2 ~ 1,
  lonely_1== 3 ~ 0,
  lonely_1== 4 ~ 0)) %>%
 mutate(isolated2 = case_when(
  isolated_2==  1 ~ 1,
  isolated_2 == 2 ~ 0,
  isolated_2 == 3 ~ 0,
  isolated_2 == 4 ~ 0)) %>%
 mutate(lonelyd2 = case_when(
  lonely_2== 1 ~ 1,
  lonely_2== 2 ~ 1,
  lonely_2== 3 ~ 0,
  lonely_2== 4 ~ 0)) %>%
 mutate(edugroup = case_when(
  edugroup_1== 1 ~ 0,
  edugroup_1== 2 ~ 0,
  edugroup_1== 3 ~ 0,
```

```
  edugroup_1== 4 ~ 1,
  edugroup_1== 5 ~ 1,
  edugroup_1== 6 ~ 2,
  edugroup_1== 7 ~ 2)) %>%
 mutate(smoker = case_when(
  smoker_1== 1 ~ 0,
  smoker_1== 2 ~ 0,
  smoker_1== 3 ~ 1,
  smoker_1== 4 ~ 1)) %>%
 mutate(bmi_who = case_when(
  bmi_1<25 ~ 0,
  bmi_1>=25 & bmi_1<30 ~ 1,
  bmi_1>= 30 ~ 2,)) %>%
 mutate(alcweekly = case_when(
  alcweekly_1== 0 ~ 0,
  alcweekly_1== 1 ~ 0,
  alcweekly_1== 2 ~ 0,
  alcweekly_1== 3 ~ 1,
  alcweekly_1== 4 ~ 1)) %>%
 # mutate(physical = case_when(
 #   physical_1>=2 ~ 0,
 #   physical_1>0 ~ 1,
 #   physical_1<2 ~ 1)) %>%
 mutate(anyill = ifelse(anyill_1 >=1 , 1, 0))%>%
 mutate(dep_l2w = ifelse(dep_l2w_1 >=18 , 1, 0)) %>%
 mutate(physical = ifelse(physical_1 >=3.6 , 1, 0)) %>%
 ungroup()
ds2 %>% frq(physical)
library(survival)
#Surv(time_dead, dead)
ds2 %>% do(tidy(coxph(Surv(time_dead, dead) ~ lonelyd, data=.), conf.int=TRUE,  exponentiate = TRUE))
ds2 %>% do(tidy(coxph(Surv(time_dead, dead) ~ isolated, data=.), conf.int=TRUE,  exponentiate = TRUE))
lltidy(coxph(Surv(time_dead, dead) ~ age+sex+lonelyd, data=ds2))
lltidy(coxph(Surv(time_dead, dead) ~ age+sex+isolated, data=ds2))
#making the anyinflammation dates and variables
#making ICD-10 diagnoses
#First the codes with varying lengths
inf1 <- c( "C46" , "D733" , "E321" , "G01" , "G02" , "G03" ,
      "G04" , "G05" , "G06" , "G07" , "H00" , "H010" ,
      "H050" , "H061" , "H100" , "H105" , "H130" , "H190" ,
      "H191" , "H192" , "H220" , "H320" , "H440" , "H600" ,
      "H601" , "H602" , "H603" , "H620" , "H621" , "H622" ,
      "H623" , "H623" , "H660" , "H670" , "H671" , "H700" ,
      "H750" , "I301" , "I320" , "I321" , "I330" , "I400" ,
      "I410" , "I411" , "I421" , "I430" , "I520" , "I521" ,
      "I681" , "J010" , "J02" , "J020" , "J028" , "J029" ,
      "J03" , "J030" , "J038" , "J039" , "J04" , "J051" ,
      "J09" , "J1" , "J2" , "J36" , "J390" , "J391" , "J851" ,
      "J852" , "J853" , "J86" , "K02" , "K044" , "K045" ,
      "K046" , "K047" , "K050" , "K052" , "K053" , "K054" ,
      "K113" , "K122" , "K230" , "K231" , "K35" , "K570" ,
      "K572" , "K574" , "K578" , "K61" , "K630" , "K650" ,
      "K670" , "K671" , "K672" , "K673" , "K678" , "K750" ,
      "K770" , "L0" , "M00" , "M01" , "M462" , "M463" , "M465" ,
      "M490" , "M491" , "M492" , "M493" , "M600" , "M630" ,
      "M631" , "M632" , "M650" , "M651" , "M710" , "M711" ,
      "M726" , "M730" , "M731" , "M86" , "N080" , "N10" ,
```

```
        "N136" , "N151" , "N160" , "N290" , "N291" , "N300" ,
        "N340" , "N390" , "N410" , "N412" , "N45" , "N61" ,
        "N700" , "N710" , "N730" , "N733" , "N74" , "N751" ,
        "N760" , "N764" , "N770" , "N771" , "O030" , "O035" ,
        "O040" , "O045" , "O050" , "O055" , "O060" , "O065" ,
        "O070" , "O075" , "O080" , "O230" , "O231" , "O232" ,
        "O233" , "O234" , "O235" , "O239" , "O753" , "O85" ,
        "O86" , "O86" , "O910" , "O911" , "O98" , "P23" , "P3" ,
        "R572" , "R650" , "R651" , "Z21")
  dinf1 <- dsos0 %>% filter(PDGO %in% inf1)
   koe1<-dsos0 %>% filter(substr(PDGO,1,1) == "A")
  koe2<-dsos0 %>% filter(substr(PDGO,1,1) == "B")
  koe3bb<-rbind(koe1, koe2, dinf1)
  dinf3bb<-koe3bb %>% group_by(id2) %>%
    arrange(id2, r_date) %>%
    filter(row_number()==1) %>%
    mutate(anyinf= 1) %>% mutate(infdate=r_date) %>% select(id2, anyinf, infdate, inf_diagnose = PDGO)
  dsos0c<-dsos0 %>% group_by(id2) %>% distinct(id2, .keep_all = TRUE)
  ff <- left_join(dsos0c,dinf3bb, by = "id2")
  ff <- ff %>% mutate(anyinf= if_else(is.na(anyinf), 0,anyinf) )
  ff <- ff %>% mutate(infdate= if_else(is.na(infdate),as.Date(ymd("2012-12-31")),infdate) )
  ff <- ff %>% select(id2, anyinf, infdate, inf_diagnose)
  fff <- merge(ds2, ff, by = c("id2"))
  fff <- fff %>% mutate(infdate= if_else(dead==1,death_date,infdate) )
  fff$time_anyinf <- as.numeric(difftime(fff$infdate, as.Date(ymd("2002-12-31")), units = "weeks")/52)

  fff %>% do(tidy(coxph(Surv(time_anyinf, anyinf) ~ lonelyd, data=.), conf.int=TRUE,  exponentiate = TRUE))
  fff %>% do(tidy(coxph(Surv(time_anyinf, anyinf) ~ isolated, data=.), conf.int=TRUE,  exponentiate = TRUE))
  fff$sex<-fct_relevel(fff$sex, "NAINEN", "MIES")
  fff <- fff %>% filter(time_anyinf>0)
  fff<- fff[!with(fff,is.na(lonelyd) & is.na(isolated)),]
#  24057-23351
#  23351-18468
   fff5 <- fff
  fff5 <- within(fff5, {
    sex <- factor(sex, labels = c("Female", "Male"))
    age_group <- factor(age_group, labels = c(" -49", "50 - 59", "60 - "))
    edugroup  <- factor(edugroup, labels = c("Lower", "Intermediate", "Higher"))
    anyill  <- factor(anyill, labels = c("No", "Yes"))
    physical  <- factor(physical, labels = c("Low", "High"))
    smoker<- factor(smoker , labels = c("No", "Yes"))
    alcweekly  <- factor(alcweekly, labels = c("Lower", "Higher"))
    dep_l2w  <- factor(dep_l2w, labels = c("Low","High"))
    # dep_l2wb  <- factor(dep_l2wb, labels = c("Low", "High"))
    isolated<- factor(isolated, labels = c("No", "Yes"))
    lonelyd<- factor(lonelyd, labels = c("No", "Yes"))
    #retired<- factor(retired, labels = c("No", "Yes"))
    #crp_over<- factor(crp_over, labels = c("No", "Yes"))
    anyinf<- factor(anyinf, labels = c("No", "Yes"))
    bmi_who<- factor(bmi_who, labels = c("Normal", "Over_weight", "Obese"))
  })
  table(fff5$sex)
  library(finalfit)
  library(table1)
  mydtb <- fff5 %>% select(
    "Age_years" = age,
    "Age_groups" =age_group,
```

```
    "Sex" = sex,
    "Education" = edugroup,
    #  "Retired" = retired,
    "Long_term_illness" = anyill,
    "Physical_activity" = physical,
    "Current_smoker" = smoker,
    "Alcohol_consumption" = alcweekly,
    "Depressive_mood" = dep_l2w,
    #  "Social_deprivation_index" = deprivation_index,
    #  "Cognitive_performance" = iqscore,
    #  "CRP_over_3_mg" = crp_over,
    "Socially_isolated" =isolated,
    "Feeling_lonely" = lonelyd,
    "Any_infectious_disease" = anyinf,
    "Follow_up_time_from_baseline_years"  = time_anyinf,
    "BMI" =bmi_1,
    "BMI_WHO"=bmi_who)


  table1(~ Sex+ Age_years + Age_groups+ Education+
Alcohol_consumption+Current_smoker+Physical_activity+BMI+BMI_WHO+Depressive_mood+Long_term_illne
ss+ Socially_isolated + Feeling_lonely +
         Any_infectious_disease+ Follow_up_time_from_baseline_years, data=mydtb)
  table1(~ Sex+ Age_years  + Education+
Alcohol_consumption+Current_smoker+Physical_activity+Depressive_mood+Long_term_illness+
Socially_isolated + Feeling_lonely +
         Any_infectious_disease+ Follow_up_time_from_baseline_years, data=mydtb, overall="Total",
#render.missing=NULL,
       render.categorical="FREQ (PCTnoNA%)")

  #----------------------------  Main + reversed causality analyses -----------------------------#
  table(fff$lonelyd)
  fff <- fff %>% filter(time_anyinf>0)

  fff <- fff %>% filter(time_anyinf>0)
  fff <- fff %>% mutate(infej2=if_else((infdate<as.Date(ymd("2011-12-31"))), 1, 0))
  table(fff$infej2)
  sum(!is.na(fff$lonelyd))
  frq(fff$dead)
  #-person years
  pfit <- pyears(Surv(time_anyinf, anyinf) ~ 1, data=fff, scale=365.25)
  fff %>% summarise(fu_time=sum(time_anyinf))
  fff$time_anyinf



  # models
  Cox_model1 <- coxph(Surv(time_anyinf, anyinf) ~ age+sex+lonelyd, data=fff)
  Cox_model2 <- coxph(Surv(time_anyinf, anyinf) ~
age+sex+edugroup+alcweekly+bmi_1+smoker+physical+anyill+dep_l2w+lonelyd, data=fff)
  Cox_model3 <- glm(lonelyd2 ~ age+sex+infej2, data = fff, family="binomial")
  Cox_model4 <- glm(lonelyd2 ~
age+sex+edugroup+alcweekly+bmi_1+smoker+physical+anyill+dep_l2w+infej2, data = fff, family="binomial")

  ti1<- tidy(Cox_model1, conf.int=TRUE,  exponentiate = TRUE)
  ti1 <- ti1 %>%  dplyr::select(terma =term, estimatea = estimate, conf.lowa =conf.low, conf.higha = conf.high,
p.valuea = p.value) %>% slice_tail()
  ti2<- tidy(Cox_model2, conf.int=TRUE,  exponentiate = TRUE)
```

```
  ti2 <- ti2 %>%  dplyr::select(terma =term, estimatea = estimate, conf.lowa =conf.low, conf.higha = conf.high,
p.valuea = p.value) %>% slice_tail()
  ti3<- tidy(Cox_model3, conf.int=TRUE,  exponentiate = TRUE)
  ti3 <- ti3 %>%  dplyr::select(terma =term, estimatea = estimate, conf.lowa =conf.low, conf.higha = conf.high,
p.valuea = p.value) %>% slice_tail()
  ti4<- tidy(Cox_model4, conf.int=TRUE,  exponentiate = TRUE)
  ti4 <- ti4 %>%  dplyr::select(terma =term, estimatea = estimate, conf.lowa =conf.low, conf.higha = conf.high,
p.valuea = p.value) %>% slice_tail()
  ti5<-fff %>% filter(lonelyd==0) %>% do(ltidy(glm(lonelyd2 ~
age+sex+edugroup+alcweekly+bmi_1+smoker+physical+anyill+dep_l2w+infej2, data = ., family="binomial")))
  ti5 <- ti5 %>%  dplyr::select(terma =term, estimatea = estimate, conf.lowa =conf.low, conf.higha = conf.high,
p.valuea = p.value) %>% slice_tail()

  Cox_model1b <- coxph(Surv(time_anyinf, anyinf) ~ age+sex+isolated, data=fff)
  Cox_model2b <- coxph(Surv(time_anyinf, anyinf) ~
age+sex+edugroup+alcweekly+bmi_1+smoker+physical+anyill+dep_l2w+isolated, data=fff)
  Cox_model3b <- glm(isolated2 ~ age+sex+infej2, data = fff, family="binomial")
  Cox_model4b <- glm(isolated2 ~
age+sex+edugroup+alcweekly+bmi_1+smoker+physical+anyill+dep_l2w+infej2, data = fff, family="binomial")

  ti6<- tidy(Cox_model1b, conf.int=TRUE,  exponentiate = TRUE)
  ti6 <- ti6 %>%  dplyr::select(terma =term, estimatea = estimate, conf.lowa =conf.low, conf.higha = conf.high,
p.valuea = p.value) %>% slice_tail()
  ti7<- tidy(Cox_model2b, conf.int=TRUE,  exponentiate = TRUE)
  ti7 <- ti7 %>%  dplyr::select(terma =term, estimatea = estimate, conf.lowa =conf.low, conf.higha = conf.high,
p.valuea = p.value) %>% slice_tail()
  ti8<- tidy(Cox_model3b, conf.int=TRUE,  exponentiate = TRUE)
  ti8 <- ti8 %>%  dplyr::select(terma =term, estimatea = estimate, conf.lowa =conf.low, conf.higha = conf.high,
p.valuea = p.value) %>% slice_tail()
  ti9<- tidy(Cox_model4b, conf.int=TRUE,  exponentiate = TRUE)
  ti9 <- ti9 %>%  dplyr::select(terma =term, estimatea = estimate, conf.lowa =conf.low, conf.higha = conf.high,
p.valuea = p.value) %>% slice_tail()
  ti10<-fff %>% filter(isolated==0) %>% do(ltidy(glm(isolated2 ~
age+sex+edugroup+alcweekly+bmi_1+smoker+physical+anyill+dep_l2w+infej2, data = ., family="binomial")))
  ti10 <- ti10 %>%  dplyr::select(terma =term, estimatea = estimate, conf.lowa =conf.low, conf.higha =
conf.high, p.valuea = p.value) %>% slice_tail()

  new.row0 <- data.frame(terma="Loneliness -> hospital treated infection", estimatea= NA,conf.lowa =NA,
conf.higha =NA, p.valuea= NA,stringsAsFactors=F)
  new.row1 <- data.frame(terma="Hospital treated infection -> loneliness", estimatea= NA,conf.lowa =NA,
conf.higha =NA, p.valuea= NA, stringsAsFactors=F)
  new.row2 <- data.frame(terma="", estimatea= NA,conf.lowa =NA, conf.higha =NA, p.valuea=
NA,stringsAsFactors=F)
  new.row3 <- data.frame(terma="Isolation -> hospital treated infection", estimatea= NA,conf.lowa =NA,
conf.higha =NA, p.valuea= NA,stringsAsFactors=F)
  new.row4 <- data.frame(terma="Hospital treated infection -> isolation", estimatea= NA,conf.lowa =NA,
conf.higha =NA, p.valuea= NA, stringsAsFactors=F)



  ti1 <- rbind(new.row0, ti1)
  ti3 <- rbind(new.row1, ti3)
  ti6 <- rbind(new.row3, ti6)
  ti6 <- rbind(new.row2, ti6)
  ti8 <- rbind(new.row4, ti8)

  tii <- rbind(ti1, ti2, ti3, ti4, ti5, ti6, ti7, ti8, ti9, ti10)
```

```
tii

tii[1,1]<- ""
tii[2,1]<- "Age- and sex-adjusted"
tii[3,1]<- "Multivariable adjusted"
tii[4,1]<- ""
tii[5,1]<- "Age- and sex-adjusted"
tii[6,1]<- "Multivariable adjusted"
tii[7,1]<- "Excluding those with loneliness at baseline "
tii[8,1]<- ""
tii[9,1]<- ""
tii[10,1]<- "Age- and sex-adjusted"
tii[11,1]<- "Multivariable adjusted"
tii[12,1]<- ""
tii[13,1]<- "Age- and sex-adjusted"
tii[14,1]<- "Multivariable adjusted"
tii[15,1]<- "Excluding those with isolation at baseline "


tii[1,6]<- "Loneliness -> hospital treated infection"
tii[4,6]<- "Hospital treated infection -> loneliness"
tii[9,6]<- "Isolation -> hospital treated infection"
tii[12,6]<-"Hospital treated infection -> isolation"

fffl<-fff %>% filter(lonelyd==0)
table(fff$infej2)
Cox_model5<-glm(lonelyd2 ~
age+sex+edugroup+alcweekly+bmi_1+smoker+physical+anyill+dep_l2w+factor(infej2), data = fffl,
family="binomial")
fffi<-fff %>% filter(isolated==0)
Cox_model5b<-glm(isolated2 ~
age+sex+edugroup+alcweekly+bmi_1+smoker+physical+anyill+dep_l2w+factor(infej2), data = fffi,
family="binomial")

esample3<-rownames(as.matrix(resid(Cox_model3)))
esample4<-rownames(as.matrix(resid(Cox_model4)))
esample5<-rownames(as.matrix(resid(Cox_model5)))
esample3b<-rownames(as.matrix(resid(Cox_model3b)))
esample4b<-rownames(as.matrix(resid(Cox_model4b)))
esample5b<-rownames(as.matrix(resid(Cox_model5b)))

tii[1,7]<- ""
tii[2,7]<- paste(as.character(glance(Cox_model1)[1]))
tii[3,7]<- paste(as.character(glance(Cox_model2)[1]))
tii[4,7]<- ""
tii[5,7]<- paste(as.character(glance(Cox_model3)[8]))
tii[6,7]<- paste(as.character(glance(Cox_model4)[8]))
tii[7,7]<- paste(as.character(glance(Cox_model5)[8]))
tii[8,7]<- ""
tii[9,7]<- ""
tii[10,7]<- paste(as.character(glance(Cox_model1b)[1]))
tii[11,7]<- paste(as.character(glance(Cox_model2b)[1]))
tii[12,7]<- ""
tii[13,7]<- paste(as.character(glance(Cox_model3b)[8]))
tii[14,7]<- paste(as.character(glance(Cox_model4b)[8]))
tii[15,7]<- paste(as.character(glance(Cox_model5b)[8]))
```

```
tii[1,8]<- ""
tii[2,8]<- paste(as.character(glance(Cox_model1)[2]))
tii[3,8]<- paste(as.character(glance(Cox_model2)[2]))
tii[4,8]<- ""
tii[5,8]<- table(fffl[esample3,]$lonelyd2)[2]
tii[6,8]<- table(fffl[esample4,]$lonelyd2)[2]
tii[7,8]<- table(fffl[esample5,]$lonelyd2)[2]
tii[8,8]<- ""
tii[9,8]<- ""
tii[10,8]<- paste(as.character(glance(Cox_model1b)[2]))
tii[11,8]<- paste(as.character(glance(Cox_model2b)[2]))
tii[12,8]<- ""
tii[13,8]<- table(fffi[esample3b,]$isolated2)[2]
tii[14,8]<- table(fffi[esample4b,]$isolated2)[2]
tii[15,8]<- table(fffi[esample5b,]$isolated2)[2]

par(family = "Times New Roman")
rowseq <- seq(nrow(tii),1)
par(mai=c(1,0,0,0))
par(omi = c(0, 0, 0, 0)) # Outer margins back
plot(tii$estimatea, rowseq, pch=15,cex = 1.5,
    xlim=c(1,1), ylim=c(0,17),
    xlab='', ylab='', yaxt='n', xaxt='n',
    bty='n', log="x")
axis(1, round(log(seq(0.6,3,by=0.1))+1, 1), cex.axis=1.1, font=2)

segments(1,-1,1, 14, lty=3, lwd=1.5)
segments(tii$conf.lowa, rowseq, tii$conf.high, rowseq, cex=4, lwd=2)


text(0.1,(nrow(tii)+1.7), "Direction of association \nand model", cex=1.2, font=2, pos=4)
text(0.42,(nrow(tii)+1.7), "N(cases)", cex=1.2, font=2, pos=4)
text(0.33 ,(nrow(tii)+1.7), "N(total)", cex=1.2, font=2, pos=4)
text(0.8,(nrow(tii)+1.7), "Relative risk (95% confidence interval)", cex=1.2, font=2, pos=4)


t1h <- tii$terma
text(0.11,rowseq, t1h, cex=1.2, pos=4, font=1)
t2h <- tii$V6
text(0.1,rowseq, t2h, cex=1.2, pos=4, font=2)
t3b <- with(tii, paste(sprintf("%.2f",estimatea, 2),' (',sprintf("%.2f",conf.lowa, 2),' to
',sprintf("%.2f",conf.higha, 2),')',sep=''))
t3 <- gsub("(NA to NA)","", t3b)
t3 <- gsub("NA ()", "", t3)
t3 <- gsub("\\()", "", t3)
text(1.9,rowseq, t3, cex=1.2, font =1, pos=4)

t4h <- tii$V7
text(0.33,rowseq, t4h, cex=1.2, pos=4, font=1)

t5h <- tii$V8
text(0.42,rowseq, t5h, cex=1.2, pos=4, font=1)


segments(0.1, 16, 3,16   , lty=1, lwd=1.2)
```

```
dev.print(tiff , "/Users/melovain/Documents/kone/Akatemia20/UKBB_Infl/LPH_rew/Kuvat/SFigure_rew", res
= 100, height = 10, width = 17, units
        = "in", compression = "zip")
```

Additional results

Figure S3 shows findings from age- and sex-adjusted analyses of the associations between loneliness, social isolation and infectious diseases for the total cohort and subgroups in the UK Biobank. The association between loneliness and hospital treated infections did not differ between those with Caucasian ethnic background (HR 1.41, 95% CI 1.36 – 1.46) and others (HR = 1.26, 95% CI 1.11 – 1.44)(p for interaction 0.12). In contrast, the association between social isolation and hospital treated infections was stronger in those with Caucasian ethnic background (HR = 1.26, 95% CI 1.23 – 1.30) compared to others (HR 1.02, 95% CI 0.91 – 1.14) (p for interaction <0.001).

The association between loneliness and infectious diseases was also robust in other analyses. The results on the associations between loneliness, social isolation and infectious diseases after multivariable adjustments and excluding those with diseases that may affect the risk of infections are shown in **figure S4**. **Tables S2** and **S3** present multivariable adjusted associations between loneliness, social isolation and specific hospital-treated disease.

The multivariable-adjusted association between loneliness and infectious diseases was little changed after additional adjustment for social isolation (HR 1.12, 95% CI 1.07–1.17). When repeating the analyses using a single item loneliness measure, the corresponding multivariable-adjusted HR was 1.11, (95% CI 1.08–1.14). Of the participants, 81 151 (17.8%) provided an affirmative response to this item.

In **figure S5**, results from replication analyses and a test of reverse causation in the associations between loneliness, social isolation and infections are shown. Data are from the HeSSup study and replicates the main findings and additionally provide no strong evidence for reverse causation**.**

Table S4 presents findings from an outcome-wide analysis of the associations of loneliness and social isolation with broad disease categories. According to multivariable adjusted analyses, loneliness and social isolation were associated with mental and behavioural disorders, thus replicating the results from other observational studies on depression, alcohol and substance abuse disorders.[29] The associations of loneliness with diseases of the respiratory, nervous, endocrine and musculoskeletal systems were weaker, but statistically significant. These associations are in agreement with previous findings.[30,31] Social isolation was not associated with these disease categories.

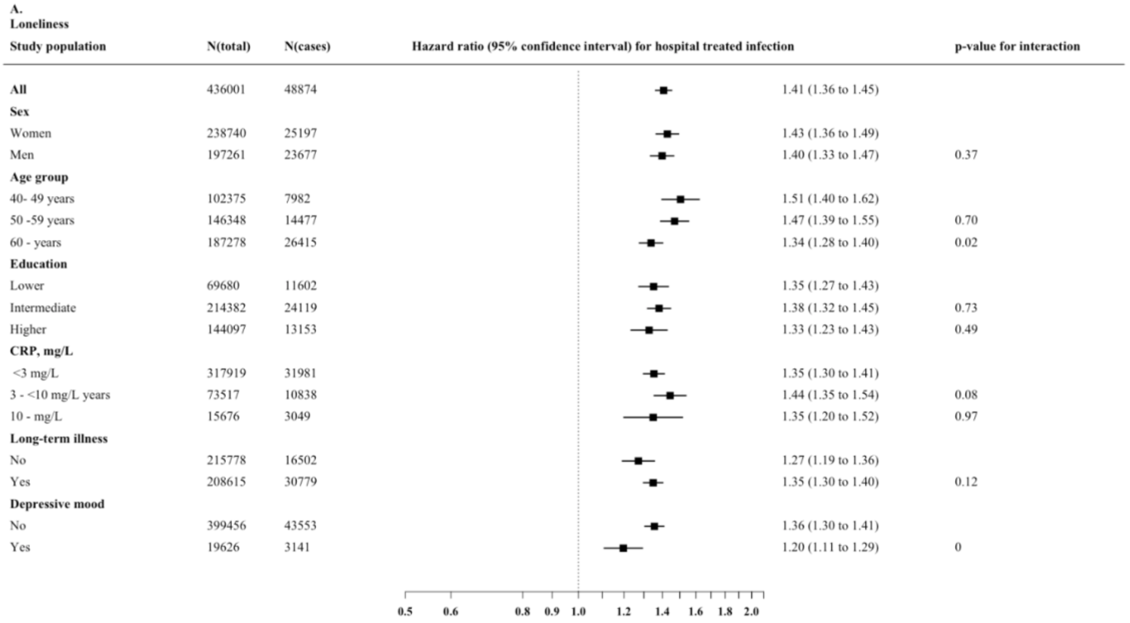

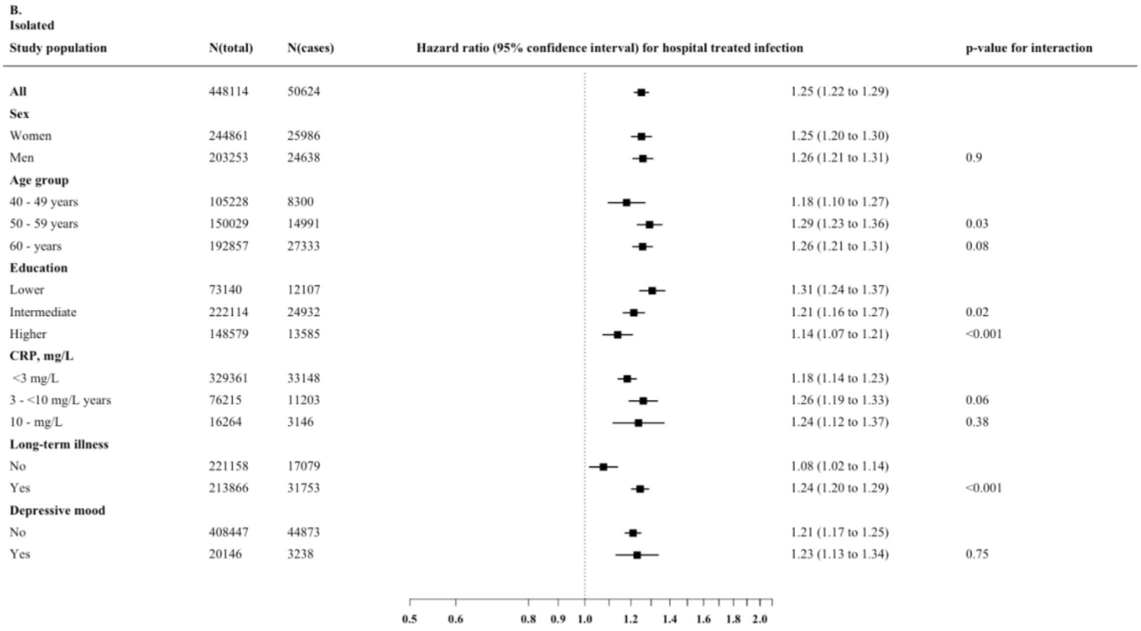

Figure S3. Associations of loneliness and social isolation with incident hospital treated infections in the UK Biobank cohort (N = 456,905) in various subgroups (adjusted for age and sex). The numbers are hazard ratios (HR) and 95% confidence intervals

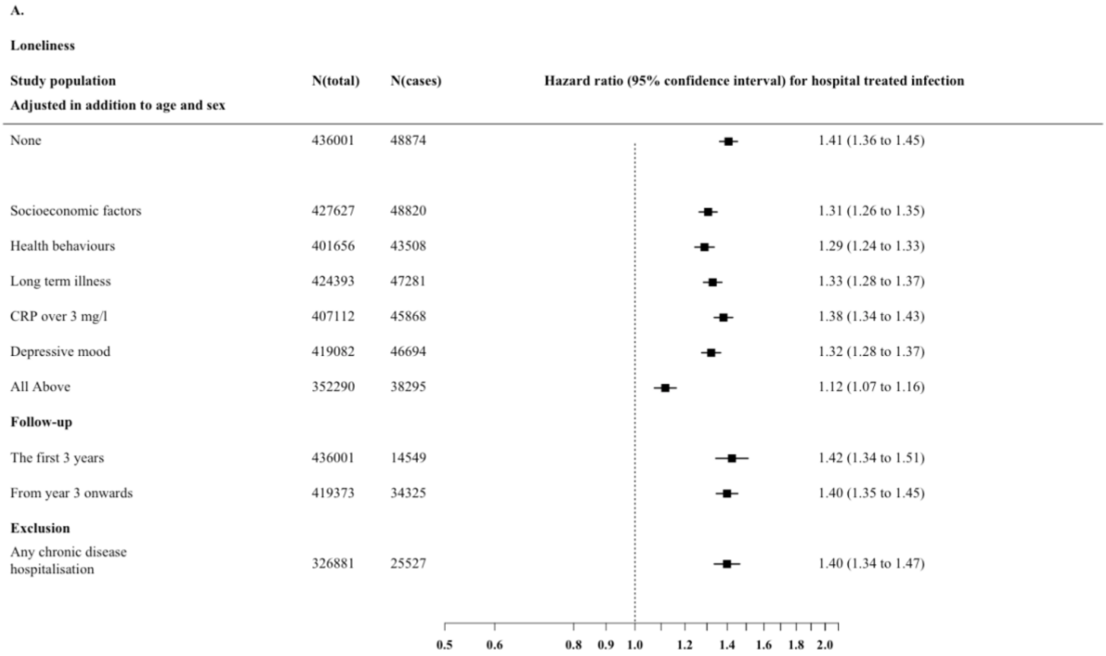

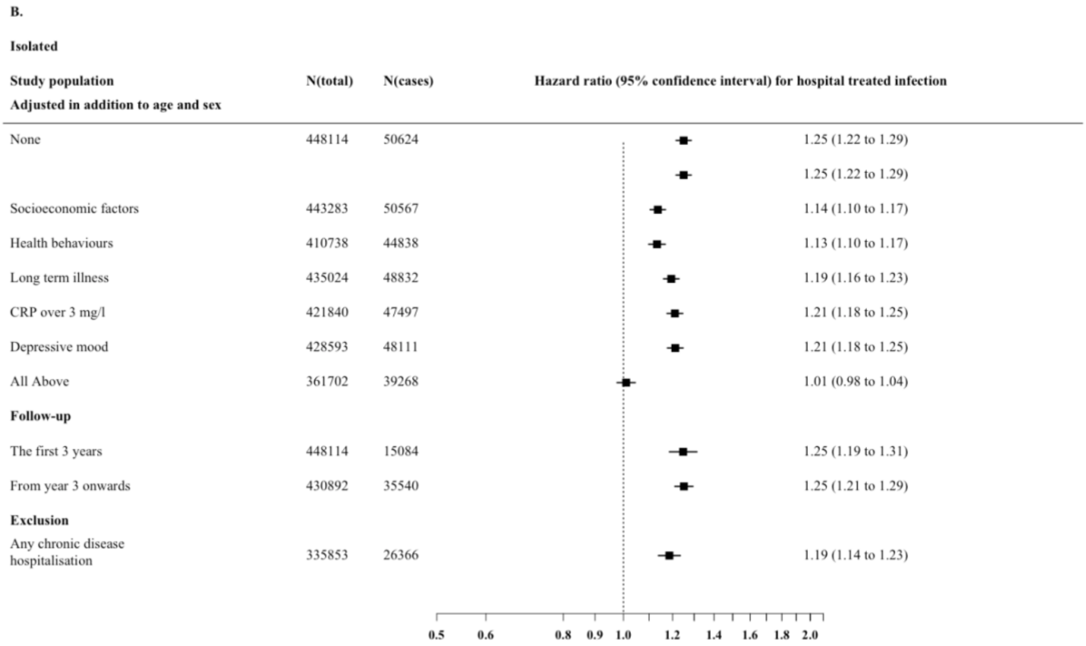

Figure S4. Associations of loneliness and social isolation with hospital treated infections in the UK Biobank cohort (N = 456,905) after multivariable adjustments, in different follow-ups, and excluding those with hospital episodes due to diseases that may affect the risk of infections (adjusted for age and sex). The numbers are hazard ratios (HR) and 95% confidence intervals

Table S2. Association (multivariable adjusted) between loneliness and risk of specific hospital-treated disease in UK Biobank cohort

N(total) 351 395	*N(cases)*	*OddsRatio*	*95%.CI*	*P-value*
Disease outcome				
Gastrointestinal infections	7713	1.12	1.02-1.22	0.02
Influenza	279	1.51	1.00-2.23	0.04
Pneumonia	6929	1.14	1.03-1.25	0.01
Urinary tract infections	7152	1.08	0.98-1.18	0.12
Skin and subcutaneous tissue infections	5181	1.07	0.95-1.19	0.26

Table S3. Association (multivariable adjusted) between isolation and risk of specific hospital-treated disease in UK Biobank cohort

N(total) 360 765	*N(cases)*	*OddsRatio*	*95%CI*	*P-value*
Disease outcome				
Gastrointestinal infections	7894	1.04	0.97-1.12	0.26
Influenza	281	0.84	0.55-1.24	0.41
Pneumonia	7102	1.12	1.03-1.20	0.01
Urinary tract infections	7303	1.06	0.98-1.15	0.11
Skin and subcutaneous tissue infections	5304	1.10	1.00-1.20	0.05

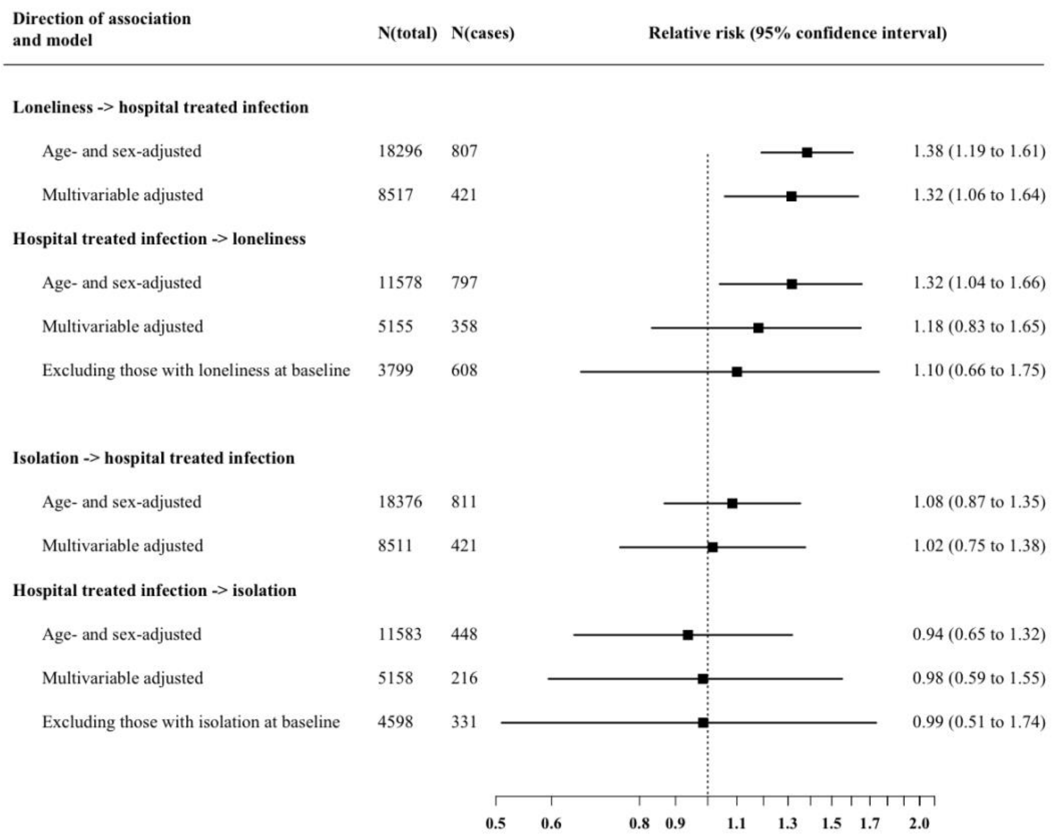

Figure S5. Multivariable-adjusted and bi-directional associations of loneliness and social isolation with hospital treated infections in the HeSSup study (N = 18,468). The numbers are hazard ratios (HR) and 95% confidence intervals (from loneliness and isolation to hospital treated infections) and Odds Ratios (OR) and 95% confidence intervals (from hospital treated infections to loneliness and isolation)

Table S4. Outcome-wide analysis of loneliness and social isolation with broad disease categories in the UK Biobank

	Exposure: Loneliness (yes vs no)			Exposure: Social isolation (yes vs no)		
	N(disease cases)	N(total)	Relative risk	N(disease cases)	N(total)	Relative risk
**Primary outcome**						
Incident infectious diseases	38 182	351 395	1.12 (1.07 to 1.16)	39 154	360 765	1.01 (0.98 to 1.05)
**Other outcomes**						
Cancer	43090	351 395	0.92 (0.88 to 0.97)	44132	360 765	0.98 (0.94 to 1.01)
Diseases of the blood	63889	351 395	1.02 (0.99 to 1.06)	65565	360 765	1.01 (0.98 to 1.04)
Endocrine diseases	68975	351 395	1.13 (1.09 to 1.17)	70783	360 765	1.01 (0.98 to 1.05)
Mental and behavioural disorders	29105	351 395	1.31 (1.25 to 1.37)	29727	360 765	1.19 (1.15 to 1.24)
Diseases of the nervous system	34567	351 395	1.13 (1.08 to 1.18)	35409	360 765	1.03 (0.99 to 1.07)
Diseases of the eye and ear	43776	351 395	1.09 (1.05 to 1.14)	44939	360 765	0.99 (0.96 to 1.03)
Diseases of the circulatory systems	113647	351 395	1.06 (1.03 to 1.10)	116675	360 765	0.97 (0.95 to 1.00)
Diseases of the respiratory systems	50284	351 395	1.14 (1.09 to 1.18)	51502	360 765	1.01 (0.98 to 1.04)
Diseases of the digestive systems	131181	351 395	1.06 (1.03 to 1.10)	134569	360 765	0.98 (0.95 to 1.00)
Diseases of the skin	32664	351 395	1.06 (1.01 to 1.11)	33457	360 765	0.98 (0.94 to 1.02)
Diseases of the musculoskeletal system	98066	351 395	1.11 (1.07 to 1.15)	100498	360 765	0.93 (0.90 to 0.95)
Diseases of the genitourinary system	84006	351 395	1.02 (0.99 to 1.06)	86114	360 765	0.97 (0.94 to 1.00)

Colour code

≤1.10
1.15
1.20
1.25
≥1.30

References

1. Perlman P. Toward a social psychology of loneliness. *Personal Relationships* 1981; **3**: 31-56.
2. Victor C, Scambler S, Bond J, Bowling A. Being alone in later life: loneliness, social isolation and living alone. *Rev Clin Gerontol* 2000; **10**: 407-17.
3. Perlman D, Peplau LA. Toward a social psychology of loneliness. *Personal relationships* 1981; **3**: 31-56.
4. Wigfield A, Turner R, Alden S, Green M, Karania V. Developing a New Conceptual Framework of Meaningful Interaction for Understanding Social Isolation and Loneliness. *Social Policy and Society* 2020: 1-22.
5. Sudlow C, Gallacher J, Allen N, et al. UK biobank: an open access resource for identifying the causes of a wide range of complex diseases of middle and old age. *PLoS Med* 2015; **12**(3): e1001779.
6. Elovainio M, Hakulinen C, Pulkki-Raback L, et al. Contribution of risk factors to excess mortality in isolated and lonely individuals: an analysis of data from the UK Biobank cohort study. *Lancet Public Health* 2017; **2**(6): E260-E6.
7. Hakulinen C, Pulkki-Raback L, Virtanen M, Jokela M, Kivimaki M, Elovainio M. Social isolation and loneliness as risk factors for myocardial infarction, stroke and mortality: UK Biobank cohort study of 479 054 men and women. *Heart* 2018.
8. Elovainio M, Lahti J, Pirinen M, et al. Association of social isolation, loneliness and genetic risk with incidence of dementia: UK Biobank Cohort Study. *BMJ Open* 2022; **12**(2): e053936.
9. Hughes ME, Waite LJ, Hawkley LC, Cacioppo JT. A Short Scale for Measuring Loneliness in Large Surveys: Results From Two Population-Based Studies. *Res Aging* 2004; **26**(6): 655-72.
10. Sarason IG, Sarason BR, Potter EH, 3rd, Antoni MH. Life events, social support, and illness. *Psychosom Med* 1985; **47**(2): 156-63.
11. Kung CSJ, Pudney SE, Shields MA. Economic gradients in loneliness, social isolation and social support: Evidence from the UK Biobank. *Soc Sci Med* 2022; **306**: 115122.
12. Statistics OfN. Testing of Loneliness Questions in Surveys. 2018, December 5. https://www.ons.gov.uk/peoplepopulationandcommunity/wellbeing/ (accessed 15 March 2022).
13. Wang S, Quan L, Ding M, et al. Depression, worry, and loneliness are associated with subsequent risk of hospitalization for COVID-19: a prospective study. *Psychol Med* 2022: 1-10.
14. LeRoy AS, Murdock KW, Jaremka LM, Loya A, Fagundes CP. Loneliness predicts self-reported cold symptoms after a viral challenge. *Health Psychol* 2017; **36**(5): 512-20.
15. Sipila PN, Heikkila N, Lindbohm JV, et al. Hospital-treated infectious diseases and the risk of dementia: a large, multicohort, observational study with a replication cohort. *Lancet Infect Dis* 2021; **21**(11): 1557-67.
16. Klein SL, Flanagan KL. Sex differences in immune responses. *Nat Rev Immunol* 2016; **16**(10): 626-38.
17. Simon AK, Hollander GA, McMichael A. Evolution of the immune system in humans from infancy to old age. *Proc Biol Sci* 2015; **282**(1821): 20143085.
18. Stead WW, Senner JW, Reddick WT, Lofgren JP. Racial differences in susceptibility to infection by Mycobacterium tuberculosis. *N Engl J Med* 1990; **322**(7): 422-7.

19. Maddison SE, Stewart CC, Farshy CE, Reimer CB. The relationship of race, sex, and age to concentrations of serum immunoglobulins expressed in international units in healthy adults in the USA. *Bull World Health Organ* 1975; **52**(2): 179-85.
20. Khan SR, van der Burgh AC, Peeters RP, van Hagen PM, Dalm V, Chaker L. Determinants of Serum Immunoglobulin Levels: A Systematic Review and Meta-Analysis. *Front Immunol* 2021; **12**: 664526.
21. Khan SR, Chaker L, Ikram MA, Peeters RP, van Hagen PM, Dalm V. Determinants and Reference Ranges of Serum Immunoglobulins in Middle-Aged and Elderly Individuals: a Population-Based Study. *J Clin Immunol* 2021; **41**(8): 1902-14.
22. Townsend JD, P. P, Beattie A. Health and Deprivation: Inequality and the North. . London: Routledge 1988.
23. Spitzer RL, Kroenke K, Williams JB. Validation and utility of a self-report version of PRIME-MD: the PHQ primary care study. Primary Care Evaluation of Mental Disorders. Patient Health Questionnaire. *JAMA* 1999; **282**(18): 1737-44.
24. Beck A, Beck R. Screening depressed patients in family practice. A rapid technic. *Postgrad Med* 1972; **52**: 81-5.
25. Mendes MA, da Silva I, Ramires V, et al. Metabolic equivalent of task (METs) thresholds as an indicator of physical activity intensity. *PloS one* 2018; **13**(7): e0200701.
26. Batty GD, Deary IJ, Luciano M, Altschul DM, Kivimaki M, Gale CR. Psychosocial factors and hospitalisations for COVID-19: Prospective cohort study based on a community sample. *Brain Behav Immun* 2020; **89**: 569-78.
27. Sipilä P, Heikkilä N, Lindbohm JV, et al. Hospital-treated infectious diseases and the risk of dementia: a large, multicohort, observational study with a replication cohort. *Lancet Infect Dis* 2021; **21**: 1557-1567.
28. Lin DY, Fleming TR, De Gruttola V. Estimating the proportion of treatment effect explained by a surrogate marker. . *Statistical Medicine* 1997; **16**: 1515-27.
29. Leigh-Hunt N, Bagguley D, Bash K, et al. An overview of systematic reviews on the public health consequences of social isolation and loneliness. *Public Health* 2017; **152**: 157-71.
30. Hackett RA, Hudson JL, Chilcot J. Loneliness and type 2 diabetes incidence: findings from the English Longitudinal Study of Ageing. *Diabetologia* 2020; **63**(11): 2329-38.
31. Jacobs JM, Hammerman-Rozenberg R, Cohen A, Stessman J. Chronic back pain among the elderly: prevalence, associations, and predictors. *Spine (Phila Pa 1976)* 2006; **31**(7): E203-7.
